# Factors affecting overall care experience for people living with rare conditions in the UK: exploratory analysis of a quantitative patient experience survey

**DOI:** 10.1186/s13023-024-03081-5

**Published:** 2024-02-19

**Authors:** Jennifer Jones, Marie Cruddas, Amy Simpson, Nick Meade, Daphnee Pushparajah, Michelle Peter, Amy Hunter

**Affiliations:** 1 Genetic Alliance UK, Creative Works, 7 Blackhorse Road, London, E17 6DS UK; 2 Department of Population Health Sciences, University of Leicester, University Road, Leicester, LE1 7RH UK; 3 Institute of Public Care, Oxford Brookes University, Harcourt Hill Campus, Oxford, OX2 9AT UK; 4 ALEXION PHARMA UK LTD, 3 Furzeground Way, Stockley Park, Uxbridge, UB11 1EZ UK; 5 North Thames Genomic Laboratory Hub, Great Ormond Street Hospital for Children NHS Foundation Trust, Level 5, Barclay House, 37 Queen Square, London, WC1N 3BH UK

**Keywords:** Rare conditions, Patient experience, Overall care, Exploratory analysis

## Abstract

**Background:**

Although individually rare, collectively, rare conditions are common and affect a large number of people and are often chronic, life threatening and affect multiple body systems; the majority of them have no effective treatment. The literature has identified many specific challenges for those living with rare conditions, however, we do not know which of these in combination are most likely to impact how someone rates their overall experience of care. The aim of this study is to do further exploratory analysis of the Genetic Alliance UK 2020 Rare Experience survey data to identify which variables are most strongly associated with respondents’ overall care experience.

**Results:**

There were strong associations between most of the selected survey variables and the overall rated experience of care variable. In the multiple linear regression only nine variables remained in the best fit model: ‘Trust and confidence in hospital staff involved in ongoing care’; ‘Satisfaction with information provided by healthcare professionals—following diagnosis’; ‘The professionals providing care work as a team’; ‘Feel care is coordinated effectively’; ‘The timing and frequency of appointments are convenient for the patient/carer/family’; ‘Whether or not there is a specific healthcare professional to ask questions of about the rare/undiagnosed condition’; ‘Experience of searching for a diagnosis’; ‘Knowledge of whether there is a specialist centre for the condition’; and ‘Number of different clinics attend for the condition’.

**Conclusions:**

Our findings indicate the challenges that play the largest part in explaining the varied experiences with rare disease healthcare in the UK for our survey respondents. These challenges should be further investigated with a broader sample of people affected by rare conditions, ideally through the implementation of a comprehensive national rare condition patient registry. Our findings highlight an important potential gap in the Framework, ‘trust and confidence in healthcare professionals’; further research is required to fully understand the foundations of trust and confidence.

**Supplementary Information:**

The online version contains supplementary material available at 10.1186/s13023-024-03081-5.

## Background

The EU definition of a rare condition is one that affects fewer than 1 in 2,000 of the population [1]; currently, there are estimated to be between 6000 and 8000 rare conditions with new ones continually being identified [2]. Although individually rare, collectively, rare conditions are common and affect a large number of people—30 million people in the EU [1] and over 3.5 million in the UK, equivalent to 1 in 17 of the population [3]. Rare conditions are often chronic, life threatening and affect multiple body systems; the majority of them have no effective treatment [1]. Dharssi et al. [4] reviewed the policies for rare diseases in 11 nations across the world (including the UK) considering five areas of key patient needs; improving coordination of care, diagnostic resources, access to treatments, patient awareness and support, and promoting innovative research. They found that rare disease plans were not being consistently implemented across the countries studied and that the patient community plays a continuing role in ensuring that rare disease care was improved through legislation and program development. In addition, a lack of experienced healthcare professionals and reliable, accessible information, are problematic [5].

The new UK Rare Diseases Framework [6], replacing the 2013 strategy [7] outlines four priority areas: 1—helping patients get a final diagnosis faster; 2—increasing awareness of rare diseases among healthcare professionals; 3—better coordination of care; and 4—improving access to specialist care, treatments and drugs. The selection of the priority areas in the UK Rare Diseases Framework was based in part on a 2019 survey, the “National Conversation on Rare Disease” [8] and on stakeholder events and discussions. Implementation plans, based on the UK Rare Diseases Framework, have been published for England [9, 10], Wales [11], Northern Ireland [12] and Scotland [13].

In 2020, Genetic Alliance UK (in a partnership agreement with Alexion, Astra Zeneca Rare Disease Unit) completed an extensive survey into the lived experiences of those who are affected by or care for someone with a rare, genetic or undiagnosed condition [14, 15]. Analysis from the quantitative elements of the survey showed that over one third (37%) rated their overall experience of care as either poor or very poor, and around half of the respondents did not believe the quality of their care had improved over the last five years with a further 20% reporting care to have got worse. The aim of this study is to do further exploratory analysis of the survey data to identify which variables are most strongly associated with respondents’ overall care experience.

Numerous studies have set out to capture the views of those affected by rare conditions, including quantitative surveys [16–19], studies using qualitative methodology [20] and mixed methods [21, 22]. Some studies focus on a particular condition whilst others include a range of conditions or, in the case of many quantitative studies, respondents are self-selecting based on identifying as being affected by any rare condition [16–19, 23, 24]. The findings from these studies show agreement around the importance of challenges such as delays in diagnosis, experiences of misdiagnoses, lack of access to appropriate information and support, lack of access to multidisciplinary healthcare, restricted access to treatments, lack of the availability of orphan drugs, difficulties in access to rehabilitation and care, loss of confidence in medical and social services and lack of knowledge amongst health and social care professionals. Patient experience surveys for any health condition typically include many different questions to encompass multiple aspects of care experience; a challenge for clinicians and policy-makers is to prioritise the most important aspects in order to target improvement efforts [25].

Although the literature has identified many specific challenges for those living with rare conditions, we do not know which of these in combination are most likely to impact how someone rates their overall experience of care. Our objective is therefore to identify the variables from the survey which are most meaningful for this data set and examine the relationships between those variables and patient perception of care without any a priori hypotheses about the existence or direction of these relationships.

## Methods

This paper analyses data from a survey run by Genetic Alliance UK in 2020 which was funded by Alexion through a partnership agreement. The aim of the 2020 survey was to collect patient experience data from those who have a rare, genetic or undiagnosed condition, and their carers.

### Survey instrument

An online survey of 102 questions was carried out in late June to early August 2020 using SurveyMonkey. The questions were based on previous surveys undertaken by Genetic Alliance UK [26, 27], other patient experience surveys (such as the Cancer Patient Experience Survey (CPES) [28]), other relevant studies (such as the CONCORD study focussing on care coordination [29]) and the anticipated focus of the 2020 UK Rare Diseases Framework. The survey instrument used a mixture of closed questions with pre-defined response categories and questions that invited open-ended, qualitative responses (see Additional file 1).

Respondents were asked to consent to taking part in the survey at the start; those that did not consent were exited from the survey. Respondents were asked for demographic information, and for details about their rare/undiagnosed condition. Some subsequent questions were filtered to be appropriate for those who had not yet received a diagnosis. Question areas focussed on the search for a diagnosis; information, awareness and the patient voice; coordination of care; access to specialist care and treatments; experiences related to research; the use of technology; overall experiences of care; and experiences due to Covid-19.

Five individuals piloted the survey: three parents of affected individuals (two with school-age children and one with an adult child), one affected adult and one social scientist. They reviewed the survey in their own time, responded to specific questions asked by the authors, and provided additional comments. Feedback was sent by email in four cases and by phone in one. The survey was refined in line with the pilot feedback.

### Survey sampling

The survey was open to anyone aged 18 or over who considered that they, or the person they care for, has a rare, genetic or undiagnosed condition and were living in the UK. Currently, no complete sample list exists of all people who consider they have a rare, genetic or undiagnosed condition so it is not possible to create a random sample; therefore, a convenience sample approach was used. No response rates are available as the link to the survey was sent via multiple overlapping distribution routes. The survey was shared widely across Genetic Alliance UK, Rare Disease UK and Syndromes Without A Name (SWAN) UK’s^1^ [30] networks and member organisations. Due to the nature of recruitment, this work is exempt from the UK National Research Ethics Service and Health Research Authority regulations.

### Data analysis

The raw data was imported into SPSS (IBM SPSS Statistics version 26) and cleaned: 31 respondents did not give consent and therefore did not start the main part of the survey, those living outside the UK (9 respondents) were also excluded. As many respondents did not complete the whole survey but still answered many questions it was decided that those who did reach question 73, which was approximately three quarters of the way through the survey, should remain in the dataset for the exploratory analysis. This meant a further 443 were excluded. Initial descriptive statistics were produced and new variables were also created. An anonymised dataset was then created which stripped out all the qualitative data as well as potentially sensitive identifiable information such as which aspects of health were affected. Wider further analysis (including the experience of searching for a diagnosis as well as the overall care experience) was carried out on the anonymised dataset of 1,020 respondents in JASP (Version 0.16.3).

#### The data

For this study, Q91 Overall care experience is the outcome variable of interest. The survey asked respondents to rate care received on a 5-level satisfaction scale; from 1 (very poor) to 5 (very good). From the remaining survey questions, 44 were selected to examine their relationship with the outcome variable (Overall care experience). These variables were selected to reflect the wide variety of issues which are faced by those affected by rare conditions based on the published literature of the experience of living with a rare condition and gathered intelligence from Genetic Alliance UK’s member organisations who on a regular basis discuss topics of relevance to the rare disease community—see Additional file 2 for the full list of included variables. Ethnicity was excluded due to there being an over-representation of people identifying as white. There is a mix of types of variable in the dataset; the majority are ordered categorical variables (ordinal), recorded on a 5-level Likert type scale or as groupings of count data (e.g. Number of clinics attended). A small number are unordered categorical (nominal) variables (e.g. Respondent’s location), and one variable is quantitative (discrete): count of the number of aspects of health mentioned—see Additional file 2 for more details.

#### Statistical analysis

In the first part of the analysis bivariate relationships between the outcome variable and explanatory variables were explored and tests of association carried out. For the second part, regression analysis was used to find the best fitting model for the survey data and to identify those variables most strongly related to the outcome variable.

##### Stage 1. Tests of association in the two-way tables

Each of the 44 individual variables was cross-tabulated with the Overall care experience variable and tested for independence. For the different data types, different test statistics are required. Kendall’s Tau-b was used for ordinal data. For nominal data Pearson’s chi-squared test was used (unless there were only two categories in which case Kendall’s Tau-b was used) and for count data a one-way Kruskal–Wallis h test (a non-parametric Analysis of Variance) was calculated—see Additional file 2.

##### Stage 2. Linear regression to identify key variables within each section of the questionnaire

For Stage 2 of the analysis, we included the variables that at Stage 1 had a significant association with the outcome variable. This identified association but did not give information about the strength of the association or how variables act in combination. Including all variables in a correlation analysis to measure and rank the strength of association was not possible as an analysis of the complete data set of valid responses to all questions would be restricted to a very small and atypical group of respondents.

A structured approach was adopted where the selected variables were assigned to their survey section and within each section a multiple linear regression was fitted with these variables to the outcome variable (Q91 Overall care experience). To maximise the number of observations in the regression model a manual stepwise selection process was used entering variables sequentially, adding predictors if the *p*-value of the regression coefficient was < 0.03 and removing predictors where *p* > 0.1. The value of 0.03 was chosen instead of the more usual 0.05 to make the selection more restrictive to reflect the exploratory nature of the analysis.

Variables were ranked according to the size of the Spearman’s rank correlation coefficient (Spearman’s rho) and entered in this order. To help determine the order of entry of the nominal variables, a binary proxy was derived from the two largest categories but the original categorical variable was entered into the model in the place determined by the correlation coefficient of the proxy variable. The criteria for inclusion of a nominal variable was a significant change (*p* < 0.03) in the R^2^ value.

In sections where there were subgroups of similar questions the process was applied in a hierarchical way, fitting a multiple regression to the variables in the sub-group and taking forward only the variables included there to the regression model for the section. This was done to enable control of the way the regression model was fitted and to avoid any potential issues with multicollinearity when fitting regression models with highly correlated explanatory variables. For example, in section 4 of the survey there is a subgroup of questions on the extent to which the respondent agrees with the statement ‘I have confidence and trust in the professionals treating me/the person I care for’. This is asked for four groups of professionals: ‘Hospital staff involved in ongoing care’, ‘Staff at the local general practice’, ‘Paramedics and staff in Emergency Departments’ and ‘Professionals working in social care.’ A model is fitted to this sub-group first and only the variables included in this model are taken forward to be tested in the regression model for section 4.

##### Stage 3. Overall regression

For the variables selected in the regression for each section in Stage 2 an overall multiple linear regression was fitted using the same approach as for Stage 2, now applied to all the remaining selected variables.

A final step was carried out where the selected nominal variables were fitted as categorical variables and in turn checked for inclusion by a significant change (*p* < 0.03) in the R^2^ value.

The response variable and the majority of the explanatory variables are ordinal, however linear regression assumes the dependent variable is continuous. While there is a great deal of debate about the use of linear regression with this type of data it was felt to be appropriate for this exploratory study which aimed to identify the key variables within this specific data set that are associated with respondent’s care experience—and not for the purpose of examining the size of the parameter estimates. In support of this approach the outcome variable Q91 Overall care experience is not skewed and the intervals can be viewed as approximately equal. In practice, studies have shown parametric methods for Likert type data are robust to violations of assumptions [31].

## Results

### Sample characteristics

In total 1503 people started the survey; after exclusions, 1,020 remained. Roughly 300 different conditions were listed by respondents in the survey, with around 200 only reported once by different individual respondents while 17 conditions were reported by nine or more respondents. About 10% of respondents mentioned living with more than one rare condition. People living with a condition made up 82% of the respondents while the other 18% were carers (90% of them female, 10% male), most of whom (72%) cared for children under 18. Respondents lived in all four nations of the UK and the number of responses within each region was generally in proportion to the regional population. See Table 1 for a detailed breakdown of the demographics (sex, age and region) of the respondents where the person answering the survey had the condition themselves, and the people cared for if a carer completed the survey. The majority of respondents identified as white (95%), with 5% identifying as mixed or multiple ethnic groups, Indian, Pakistani, other Asian backgrounds, African, Caribbean, any other Black, African or Caribbean background, Arab, or any other ethnic group. In the survey, most people with a rare condition (91%) had received a definitive diagnosis, while 9% were described as being undiagnosed (of which 40% were children and 60% were adults); both diagnosed and undiagnosed groups are included as eligible within the analysis.

**Table 1:** Sample demographics

	Person with condition answering the survey (n = 833)	Carer describing person they care for (n = 187)	Total (n = 1020)
*Sex*			
Male	144 (17%)	101 (54%)	245 (24%)
Female	680 (82%)	82 (44%)	762 (75%)
Other	3 (0.4%)	1 (0.5%)	4 (0.4%)
Prefer not to say	4 (0.5%)	1 (0.5%)	5 (0.5%)
Missing	2 (0.2%)	2 (1%)	4 (0.4%)
*Age*			
Under 18	NA	135 (72%)	135 (13%)
18–24	51 (6%)	22 (12%)	73 (7%)
25–34	120 (14%)	11 (6%)	131 (13%)
35–44	159 (19%)	4 (2%)	163 (16%)
45–54	213 (26%)	1 (0.5%)	214 (21%)
55–64	176 (21%)	4 (2%)	180 (18%)
65–74	93 (11%)	6 (3%)	99 (10%)
75 +	19 (2%)	2 (1%)	21 (2%)
Prefer not to say	2 (0.2%)	0	2 (0.2%)
Missing	0	2 (1%)	2 (0.2%)
*Region*			
North East and Cumbria	35 (4%)	6 (3%)	41 (4%)
North West of England	93 (11%)	18 (10%)	111 (11%)
Yorkshire	64 (8%)	11 (6%)	75 (7%)
East Midlands	54 (6%)	17 (9%)	71 (7%)
West Midlands	45 (5%)	13 (7%)	58 (6%)
East of England	76 (9%)	10 (5%)	86 (8%)
London	71 (9%)	13 (7%)	84 (8%)
South East of England	155 (19%)	45 (24%)	200 (20%)
South West of England	92 (11%)	21 (11%)	113 (11%)
Wales	58 (7%)	10 (5%)	68 (7%)
Northern Ireland	12 (1%)	2 (1%)	14 (1%)
Scotland	73 (9%)	20 (11%)	93 (9%)
Prefer not to say	5 (0.6%)	0	5 (0.5%)
Missing	0	1 (0.5%)	1 (0.1%)

**Fig. 1:**
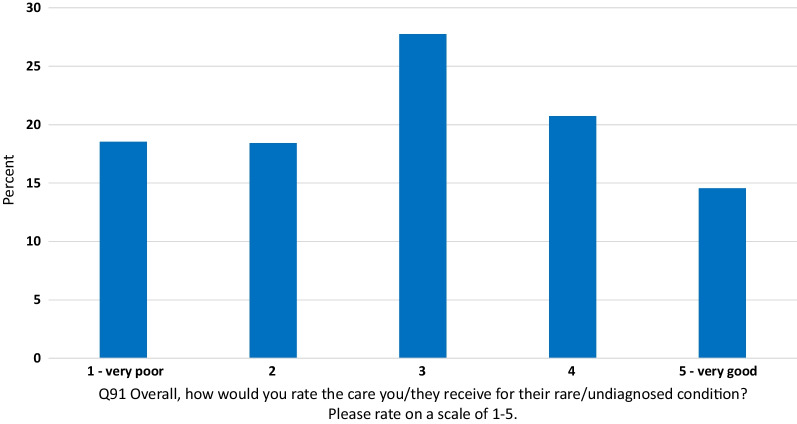
Overall care experience

The overall care rating question (Q91) indicated that just over a third of respondents (35%) rated their care as 4 or 5 out of 5 (with 5 being labelled as very good and 1 being labelled as very poor). A further 37% rated their care as 1 or 2 out of 5; 28% choose the middle care rating of 3 (see Fig. 1 and Additional file 3).

### Stage 1. Tests of association in the two-way tables

This paper reports on the part of the further exploratory analysis which focussed on the respondent’s overall care experience and aimed to identify the strengths of association with the selected variables. Of the 44 variables tested in Stage 1, most (38 variables) showed significant associations with how respondents rated their overall care experience. Table 2 shows the results broken down into themes based on the section of the survey where the question appeared. The variables that did not show an association were: (1) whether the patient was an adult or child, (2) patient’s age, (3) where the patient lived, (4) who diagnosed the condition, (5) the furthest clinic attended, and (6) how many times the respondents had contact with health services.

**Table 2:** Associations between overall care experience and selected variables

Variable/question (by survey section)	Stage 1 test statistics	n	*p*	Notes
*Section 1: Demographics*				
Age of patient	Kendall's Tau-b—**τb = *0.026***	953	*p* = *0.31*	No evidence of association
Child/Adult indicator	Kendall's Tau-b—**τb = -*0.018***	953	*p* = *0.53*	No evidence of association
Sex of patient	Kendall's Tau-b—**τb = *0.092***	945	p < 0.05	Males have a more positive overall care rating than females
Respondent’s location (UK Nation)	Chi-squared—**χ**^**2**^** = *9.883***	939	*p* = *0.27*	No evidence of association
Patient's ability to work affected	Kendall's Tau-b—**τb = *-0.235***	707	p < .001	Those not affected have a more positive overall care rating than those affected
Patient's ability to study affected	Kendall's Tau-b—**τb = *-0.214***	504	p < .001	Those not affected have a more positive overall care rating than those affected
*Section 2: About the rare/undiagnosed condition*				
Count of number of aspects of health mentioned	Kruskal–Wallis test = ***72.56***	955	p < .001	Those with fewer aspects of health affected have a more positive overall care rating than those with more aspects of health affected
Condition Complexity (Grouped count of number of aspects of health mentioned)	Kendall's Tau-b—**τb = *-0.214***	955	p < .001	Those with fewer aspects of health affected have a more positive overall care rating than those with more aspects of health affected
Whether or not diagnosed	Kendall's Tau-b—**τb = *0.12***	930	p < .001	Those with a diagnosis have a more positive overall care rating than those who are undiagnosed
Number of years since diagnosis	Kendall's Tau-b—**τb = *0.11***	825	p < .001	Those diagnosed a longer time ago have a more positive overall care rating than those diagnosed more recently
*Section 3: Diagnosis*				
Whether have been misdiagnosed	Kendall's Tau-b—**τb = *-0.262***	870	p < .001	Those who have not experienced misdiagnosis have a more positive overall care rating than those who have had a misdiagnosis
Number of times misdiagnosed	Kendall's Tau-b—**τb = *-0.239***	410	p < .001	Those with fewer misdiagnoses have a more positive overall care experience than those with more misdiagnoses
Length of wait for definitive diagnosis	Kendall's Tau-b—**τb = *-0.292***	776	p < .001	Those with a shorter wait for a definitive diagnosis have a more positive overall care rating
Which healthcare professional made the diagnosis	Chi-squared—**χ**^**2**^** = *25.11***	841	*p* = *0.07*	No evidence of association
Experience of searching for a diagnosis	Kendall's Tau-b—**τb = *0.458***	943	p < .001	Those with a more positive experience of searching for a diagnosis have a more positive overall care rating
*Section 4: Information, awareness and the patient voice*				
Satisfaction with information provided—before diagnosis	Kendall's Tau-b—**τb = *0.363***	851	p < .001	Those who are more satisfied have a more positive overall care rating
Satisfaction with information provided—at diagnosis	Kendall's Tau-b—**τb = *0.385***	849	p < .001	Those who are more satisfied have a more positive overall care rating
Satisfaction with information provided—following diagnosis	Kendall's Tau-b—**τb = *0.554***	851	p < .001	Those who are more satisfied have a more positive overall care rating
Agreement with statement ' I have sufficient information about condition'	Kendall's Tau-b—**τb = *0.335***	952	p < .001	Those who agreed more strongly with the statement have a more positive overall care rating
Is there a specific heath care professional to contact about the condition?	Kendall's Tau-b—**τb = *0.491***	884	p < .001	Those who had a specific healthcare professional to contact had a more positive overall care rating
If there is a specific healthcare professional to contact, how easy is it to contact them	Kendall's Tau-b—**τb = *0.388***	531	p < .001	Those who found it easier to contact the specific healthcare professional had a more positive overall care experience
Involved in decision about treatment?	Kendall's Tau-b—**τb = *0.291***	889	p < .001	Those who were involved in decisions about treatment had a more positive overall care experience
Whether or not have an alert card	Kendall's Tau-b—**τb = *0.156***	935	p < .001	Those who had an alert card had a more positive overall care experience
Trust and confidence in Hospital staff	Kendall's Tau-b—**τb = *0.598***	879	p < .001	Those who agreed more strongly with the statement have a more positive overall care rating
Trust and confidence in GP staff	Kendall's Tau-b—**τb = *0.265***	918	p < .001	Those who agreed more strongly with the statement have a more positive overall care rating
Trust and confidence in ED staff and Paramedics	Kendall's Tau-b—**τb = *0.288***	761	*p* < *.001*	Those who agreed more strongly with the statement have a more positive overall care rating
Trust and confidence in Social care staff	Kendall's Tau-b—**τb = *0.352***	562	p < .001	Those who agreed more strongly with the statement have a more positive overall care rating
Hospital staff are sufficiently informed about condition	Kendall's Tau-b—**τb = *0.466***	871	p < .001	Those who agreed more strongly with the statement have a more positive overall care rating
GP staff are sufficiently informed about condition	Kendall's Tau-b—**τb = *0.285***	908	p < .001	Those who agreed more strongly with the statement have a more positive overall care rating
Paramedics are sufficiently informed about condition	Kendall's Tau-b—**τb = *0.291***	755	p < .001	Those who agreed more strongly with the statement have a more positive overall care rating
Social care staff are sufficiently informed about condition	Kendall's Tau-b—**τb = *0.322***	577	p < .001	Those who agreed more strongly with the statement have a more positive overall care rating
*Section 5: Coordination of care*				
Number of times had contact with Health Service (Grouped)	Kendall's Tau-b—**τb = *0.027***	868	p = 0.32	No evidence of association
Number of different clinics attend	Kendall's Tau-b—**τb = *0.150***	943	p < .001	Those who attend more clinics have a more positive overall care experience than those who attend fewer clinics
Time to travel to furthest clinic	Kendall's Tau-b—**τb =*− 0.004***	803	p = 0.88	No evidence of association
Who coordinates care?	Chi-squared—***χ***^***2***^** = *89.168***	909	p < .001	Those who have a dedicated coordinator/or shared responsibility have a more positive overall care experience than those who do their own care-coordination
Whether have a care plan	Kendall's Tau-b—**τb = *0.121***	875	p < .001	Those who have a care plan had a more positive overall care experience
Feel that care is coordinated effectively	Kendall's Tau-b—**τb = *0.567***	753	p < .001	Those who feel care is coordinated effectively have a more positive overall care experience than those who do not feel care is effectively coordinated
Agreement with: The professionals providing care work as a team	Kendall's Tau-b—**τb = *0.549***	825	p < .001	Those who agreed more strongly with the statement have a more positive overall care rating
Agreement with: The timing and frequency of appointments are convenient	Kendall's Tau-b—**τb = *0.472***	848	p < .001	Those who agreed more strongly with the statement have a more positive overall care rating
Agreement with: Would prefer more appointments to be provided locally	Kendall's Tau-b—**τb = *− 0.186***	746	p < .001	Those who agreed less strongly with the statement have a more positive overall care rating
*Section 6: Access to specialist care and treatments*				
Do they have a doctor who is an expert?	Kendall's Tau-b—**τb = *0.464***	880	p < .001	Those who have a doctor who is an expert have a more positive overall care rating than those who do not have an expert doctor
Whether there is a specialist centre (Yes/No/unsure)	Chi-squared—***χ***^***2***^** = *74.008***	955	p < .001	Those who were aware that there was a specialist centre for their condition had a more positive overall care experience than those who were not aware of a specialist centre
If so whether they access the specialist centre	Kendall's Tau-b—**τb = *0.486***	439	p < .001	Those who accessed a specialist centre had a more positive care experience than those who did not
*Section 9: Overall care*				
Changes in quality of care in past 5 years (it has got worse, it has stayed the same, it has improved)	Kendall's Tau-b—**τb = *0.260***	818	p < .001	Those who had a better quality of care had a more positive care experience than those who had a worse quality of care

### Stage 2. Linear regression to identify key variables

The second stage of the analysis started with the remaining 38 variables from Stage 1; a further two variables (Q48 and Q74) were excluded at this point as they were only asked or relevant to a subsection of the sample (see Additional file 3). Regression analyses were then performed on the variables within each section of the questionnaire separately to get a ‘best’ model for each section. The variables selected in each model then became the candidate variables for the overall regression (results not shown); 23 variables remained at the end of Stage 2.

### Stage 3. Variables in the best fit model

The Stage 1 analysis showed strong associations between most of the variables and the rated experience of care. After excluding variables as described in Stages 1 and 2 of the analysis and systematically fitting the remaining variables in a multiple linear regression only nine variables were ultimately included in the best fit model (see Table 3):

1. Trust and confidence in hospital staff involved in ongoing care
2. Satisfaction with information provided by healthcare professionals—following diagnosis
3. The professionals providing care work as a team
4. Feel care is coordinated effectively
5. The timing and frequency of appointments are convenient for the patient/carer/family
6. Whether or not there is a specific healthcare professional to ask questions of about the rare/undiagnosed condition
7. Experience of searching for a diagnosis
8. Knowledge of whether there is a specialist centre for the condition—coded as No, Yes, Unsure
9. Number of different clinics attend for the condition—coded as 0, 1–2, 3–4 and 5 +

**Table 3:** Variables included in the best-fit model for overall care experience

Variable	Category	Estimate	Standard Error	*p*
*Intercept*		0.81	0.17	< .001
Trust and confidence in Hospital staff	Strongly disagree (reference)			
	Disagree	0.21	0.14	0.14
	Neither agree nor disagree	0.44	0.16	0.01
	Agree	0.69	0.15	< .001
	Strongly agree	0.85	0.16	< .001
Satisfaction with information provided by healthcare professionals—following diagnosis	Very unsatisfied (reference)			
	Unsatisfied	0.15	0.13	0.24
	Neither satisfied nor unsatisfied	0.30	0.14	0.03
	Satisfied	0.49	0.14	< .001
	Very satisfied	0.73	0.15	< .001
The professionals providing care work as a team	Strongly disagree (reference)			
	Disagree	0.13	0.12	0.26
	Neither agree nor disagree	0.09	0.13	0.52
	Agree	0.32	0.15	0.03
	Strongly agree	0.70	0.19	< .001
The timing and frequency of appointments are convenient for the patient/carer/family	Strongly disagree (reference)			
	Disagree	0.06	0.13	0.66
	Neither agree nor disagree	0.16	0.13	0.23
	Agree	0.35	0.14	0.01
	Strongly agree	0.46	0.18	0.01
Experience of searching for a diagnosis	1—I feel/felt abandoned by the NHS (reference)			
	2	0.31	0.12	0.01
	3	0.54	0.11	< .001
	4	0.41	0.13	< .005
	5—The NHS never gave up/has not given up	0.56	0.11	< .001
Knowledge of whether there is a specialist centre for the condition	No (reference)			
	Yes	0.28	0.09	< .005
	Unsure	0.19	0.11	0.08
Number of different clinics attend for the condition	None (reference)			
	1–2	−0.02	0.13	0.89
	3–4	−0.05	0.14	0.75
	5 +	0.33	0.15	0.02
Feel care is coordinated effectively	No (reference)			
	Yes	0.29	0.12	0.01
Is there a specific healthcare professional to ask questions about the rare condition?	No (reference)			
	Yes	0.31	0.09	< .001

Due to missing data on one or more variables, the final model is fitted to 525 of the observations. The model accounted for just over two thirds (68%—R^2^ value) of the total variance in overall care experience.

The residuals from the final model were examined to assess how well they complied with the assumptions required for fitting linear regression models. Plots of the distribution of the standardised residuals and of the ordered standardised residuals against the theoretical quantiles (the Q–Q plot) gave a good indication that residuals are normally distributed. The Durbin-Watson statistic = 2.028 with *p* = 0.76, indicates that the residuals are not auto-correlated and that the assumption of independence is met. To examine the assumption of constant variance in the error term (homoscedasticity) we plotted the residuals against the predicted values. The plot showed a pattern of parallel lines which occurs because as previously noted we are treating a variable that can only take the values 1–5 as continuous and so the points will not be randomly scattered. However, the plot does not provide evidence of heteroscedasticity as there is no clear curvature and there is a constant spread across the predicted values. Given this evidence of compliance with the assumptions and the discussion in the previous section it was felt that the use of linear regression was appropriate for this analysis.

Four of the nine variables consider the level of agreement or satisfaction with a statement; in all cases the more someone is satisfied or agrees with the statement, the higher their rating of overall care. For three of these four variables (‘Having confidence and trust in hospital staff involved in ongoing care’, ‘Level of satisfaction with information provided by healthcare professionals following a diagnosis’ and ‘The timing and frequency of appointments are convenient for the patient/carer/family’) every successive positive response has a larger estimated coefficient compared with the base value of strongly disagree/very unsatisfied; indicating that the more someone was satisfied or agreed with these statements then the higher was their rating of overall care—see Table 3. For the variable ‘Rating the experience of searching for a diagnosis’ all the more favourable categories are significantly different to the zero category (I felt/feel abandoned by the NHS); so those that had a better experience of searching for a diagnosis, had higher ratings for their overall care.

For the variable ‘Q60 How many different types of clinics do you/they currently attend for your/their rare/undiagnosed condition?’, only those who attended 5 or more clinics are significantly different to the base category of attending no clinics, indicating that those who attend many different clinics have a better overall care experience.

The respondents who said that there was a specific healthcare professional who they could go to with questions about their condition had a more positive overall care experience compared with those who did not. Those who said that they felt that their care was coordinated effectively had a more positive overall care experience compared with those who did not feel that their care was effectively coordinated. For the variable ‘Q73 Do you know if there is a specialist centre for your/their condition?’ only those who said ‘Yes’ as opposed to unsure, were significantly different to the base category of ‘No’, suggesting that those with rare conditions who are aware there is a specialist centre have a more positive experience of care overall.

## Discussion

**Fig. 2:**
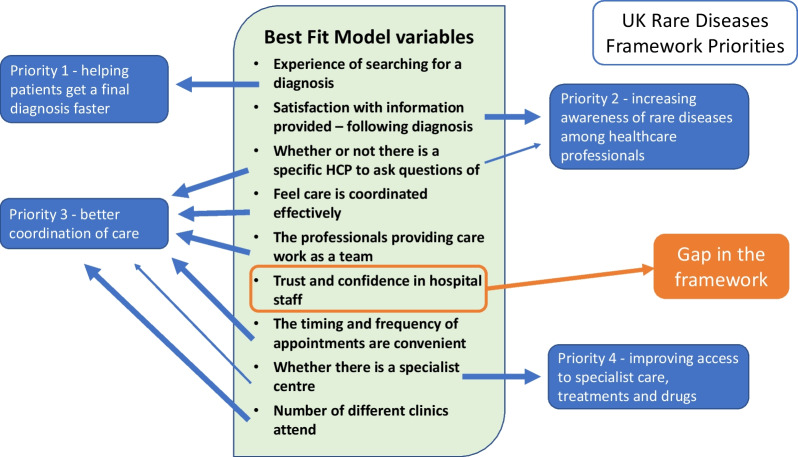
How the nine best fit model variables could fit under each of the four priorities

Many specific challenges for those with rare conditions have been identified and the purpose of this study was to go beyond individual variables to better understand which combination of variables is the strongest for indicating good and poor experiences of overall care. This is a key first step in order to ultimately focus effort on the most effective service improvements. Most of the variables analysed individually in our study do have a significant association with how respondents rated their overall care experience, which is not surprising as the variables in the survey reflected known issues faced by those affected by rare conditions, however, the best fit model included only nine of these 44 variables which in combination best explained overall care variance (see Fig. 2).

Eight of the nine variables align with the four priority areas of the UK Rare Diseases Framework with some variables falling under more than one priority area. For example, ‘Whether there is a doctor to ask questions of’ could be included under priority 2 (Increasing awareness of healthcare professionals) and priority 3 (Care coordination), see Fig. 2.

We describe below how each of the nine best fit model variables identified in this study map onto the four priority areas from the UK Rare Diseases Framework. As the predominant policy vehicle driving progress in rare diseases in the UK, and one that is familiar to UK stakeholders, the Framework provides a useful structure for discussing our findings.

### Priority 1—helping patients get a final diagnosis faster

#### Survey question/variable: experience searching for a diagnosis

Our best fit model shows that the journey of looking for a diagnosis (often referred to as the diagnostic odyssey) plays a role in how people rate their overall care experience: the better the experience of searching for a diagnosis, the higher respondents rated their overall care experience. The qualitative findings from the Genetic Alliance UK 2020 survey supported the importance of receiving a diagnosis for example in order to get further medical support as well as other types of support [14, 15]. People who had a negative experience of getting a diagnosis rated their care as worse. Previous research has also identified the negative impacts of diagnostic delay [32–37]. Delays have been identified as being due to slow referrals, being discharged back to the GP, limited access to genetic testing and the time taken to receive results—as well as not being viewed holistically by the healthcare professionals that people came into contact with [14, 38, 39]. Other studies have also shown negative impacts such as frustration when no diagnosis is found [40].

Our work supports the priority given to faster diagnosis by the Framework. However, as some people will never receive a diagnosis, consideration should be given not just to the speed of diagnosis but also to the wider experience of searching for and receiving a diagnosis [38, 41].

### Priority 2—increasing awareness of rare diseases among healthcare professionals

#### Survey question/variable: satisfaction with information provided by healthcare professionals following diagnosis

Less than a third (31%) of respondents in our survey were satisfied or very satisfied with the information provided before diagnosis, compared with 59% satisfied with the information provided at the point of diagnosis and around half (49%) satisfied with information provided after diagnosis—see Additional file 3. We found that being more satisfied with the information provided before, during or after diagnosis were each individually strongly associated with overall care experience. However, only the satisfaction with information provided after diagnosis remained in the best fit model. Previous research has shown that it is important that those with rare conditions as well as parents/carers, and healthcare professionals, have access to information so that a partnership can be formed between them [42]. Information about the rare condition itself as well as healthcare-related information such as available specialists and specialised services have been identified as important factors for those with rare conditions [5, 43]. Our study indicates that after diagnosis is a key time to provide information and healthcare professionals should be guided to where trusted sources of information are available about the rare condition as well as signposting to sources of support, whilst still considering what information should be provided before and during diagnosis.

### Priority 3—better coordination of care

#### Survey question/variable: whether there is a specific healthcare professional to ask questions of

Although many respondents had a specific healthcare professional they could ask questions of, not all respondents had a ‘point of contact’ within the healthcare system who could provide advice when needed, yet having a point of contact impacted positively on overall care experiences. From the open text comments within the survey, it was clear that respondents valued seeing someone who was knowledgeable about their condition [14, 15]. Respondents in the Genetic Alliance UK study wanted to be able to access reliable, up to date information and they often found this within the third sector (charities, voluntary and community groups) rather than from within the healthcare system [14, 38]. In line with other work [44], we propose that having a dedicated point of contact within the healthcare system could help parents/carers and those with a rare condition as they adjust to the diagnosis, remain undiagnosed, and receive ongoing treatment/management for their condition.

#### Survey question/variable: professionals providing care work as a team

The more strongly respondents agreed with the statement that the ‘Professionals providing care work as a team’, the more positively they rated their overall experience of care. A third (34%) of respondents agreed or strongly agreed that professionals providing their care work as a team (see Additional file 3)**.** In the CONCORD study, *“Ways of organising professionals involved in care*” was one of the six domains found to describe care coordination [45]. The importance of teamwork for the benefit of patients has been recognised but most research has only been conducted with clinical staff [46]. Zajac et al. [46] have developed frameworks aimed at all types of healthcare teams including administrative and research teams addressing the numerous challenges faced such as “*accountability, conflict management, decision-making, reflecting on progress, and coaching*”. Designing healthcare systems around team work and interaction between departments could improve the overall care experience of those with rare conditions.

#### Survey question/variable: timing and frequency of appointments are convenient for the patient/carer/family

The more strongly that respondents agreed with the statement that the ‘Timing and frequency of the appointments were convenient for the patient/carer/family’, the higher their rating of overall care experience. Similarly, for some cancer patients on certain pathways the length of waiting time for clinics and appointments is a substantial predictor of overall satisfaction for cancer care [25]. From the Genetic Alliance UK survey, respondents described various impacts of the timing and frequency of appointments voicing disappointment with having long gaps between appointments and the frustrations of having to chase up appointments [14]. Survey respondents also raised the inconvenience of having multiple appointments in multiple different settings with often little communication between healthcare professionals in the different locations. As every person affected by a rare condition has different requirements it is suggested that the timing, frequency and location of appointments is discussed with them so that the appointments can be planned to be as convenient as possible—a dedicated care coordinator could help facilitate this.

#### Survey question/variable: number of different clinics attend for the condition

The number of different clinics attended and the level of use of health care services varied greatly amongst the respondents. Those who reported attending five or more different clinics gave a better rating of overall care than those who attended no clinics. This could perhaps be explained by the respondents who visit numerous clinics feeling that their care needs are being more fully addressed through seeing different specialists.

#### Survey question/variable: feel that care is effectively coordinated

Believing that care was coordinated effectively was strongly associated with a better care experience for the Genetic Alliance UK sample. In the survey, less than a third (30%) felt their care was effectively coordinated. Those living with a rare condition as well as carers (many of whom were parents) have outlined previously how challenging it can be to coordinate care [45, 47–51]. Our findings highlight that having a dedicated care coordinator could improve the overall care experience for those with rare conditions.

### Priority 4—improving access to specialist care, treatment and drugs

#### Survey question/variable: whether there is a specialist centre

Having a specialist centre available had a positive impact on overall care experience. Specialist centres exist for only a limited number of rare conditions but data are not available to indicate how many people these specialist centres may serve. In our sample almost half (48%) stated that there was a specialist centre available for their condition. Just over half (52%) of these respondents actually attended the specialist centre which was available for their condition (see Additional file 3). Reasons for not accessing specialist centres included distance, long waiting lists or the centre only offering diagnostic services rather than ongoing care [14].

### Gaps in the UK rare disease framework

#### Survey question/variable: trust and confidence in hospital staff involved in ongoing care

In our study, those who reported having trust and confidence in hospital staff also reported better overall care. However, not all respondents felt this way: having trust and confidence in hospital staff who were involved with ongoing care was only reported by just over half (59%) of respondents (see Additional file 3). This compares poorly to what has been achieved with cancer care in healthcare systems in other countries [52].

Mistrust in doctors and the healthcare system, feelings of insecurity, and fear and anger by those with rare conditions has been reported before [53–55]. Qualitative findings from the Genetic Alliance UK survey indicated that information from and communication with healthcare professionals was sometimes poor and could lead to respondents losing faith in the system or certain healthcare providers within it, and for some respondents this potentially led to an avoidance of healthcare settings altogether [14, 38]. Distrust as a barrier to service use has also been described in other work [56]; race/ethnicity has been shown to be significantly associated with levels of institutional trust and experiences of healthcare which can lead to health disparities in certain populations [57–59]. Some respondents in the Genetic Alliance UK survey described feeling unsafe or fearful if they were in a healthcare setting with healthcare professionals who lacked knowledge of their condition [14]. Previous research has described how healthcare professionals’ aptitude to treat has been questioned when there is uncertainty regarding their medical knowledge of the rare condition [60–62].

Trust and confidence in hospital staff involved in ongoing care is not explicitly highlighted in the structure of the UK Rare Diseases Framework [6]. It is therefore unlikely to receive the attention it deserves as the four home nations implement their individual plans of action [10–13]. It might be predicted that bringing about improvements under the priorities as they are written will naturally lead to better trust and confidence—but without better insight into people’s interpretation of trust and confidence, and how this translates into perception of care, this would be a dangerous assumption. As well as improving our understanding of trust and confidence from the view of patients, we also need to explore how prepared and confident professionals feel when encountering rare conditions for the first time and having to explain these to patients establishing what support professionals feel they need to deliver the individualised high-quality care set out in the framework. Future research to further investigate ‘trust and confidence’ in healthcare professionals and how that is understood by those with rare conditions, their carers and healthcare professionals, is needed in order to guide service improvements.

Evaluation of the impact of the Framework and implementation plans is critical and requires the development of suitable metrics with input from a broad range of people affected by rare conditions. As they grow, national registration services should provide data (both clinical and experiential) to help measure the impact of implementation plans and how people with rare conditions experience care in the UK. This will highlight successful approaches and help identify when and how health services should improve overall experiences of care.

### Limitations

The best fit model does not explain all the variation in how experiences in care are rated by those with rare conditions or those caring for them. Other variables could have proved important had the survey allowed for their inclusion such as the psychological and emotional impact of having a rare condition or variables related to accessing medicines and treatment. Our qualitative data indicates that some respondents felt frustrated at the pace of drug development, or access to existing drugs or treatment, which may lead to a poorer overall experience of care [14, 15]. The availability of variables was dependent on the questions in the original survey most of which reflect areas which are known to be relevant to those living with rare conditions– this was heavily influenced by previous research with those affected by rare conditions [26, 27] and knowledge of relevant UK rare condition policies [7]. The individual questions in the survey have not been tested for validity which may limit the interpretation of some of the findings.

Although the survey focussed predominantly on healthcare, when answering the question about experiences of overall care, it is possible that respondents may have also included their experiences with social care; this should be taken into consideration when interpreting the results as there were few variables that collected data about social care experiences.

The survey took place during the Covid19 pandemic which may have influenced responses, although at the time of the survey (summer 2020) the UK was not in lockdown. While many respondents are likely to have still been shielding due to their condition, most survey questions related to experiences over several years rather than the time of survey completion. The sample is not random and there will be some sampling bias. The survey was only available online so relies on internet access which may mean that those experiencing digital poverty were not able to take part in the survey. The most notable bias is the balance of females versus males taking part in the survey and the relatively low proportion of carers who answered the survey. There is also a bias towards those who identify as white and those respondents who received a diagnosis, with potentially a bias towards those conditions where specialist centres are available; for all the groups who are under-represented in our study it is possible that their experiences of having a rare condition or caring for someone with a rare condition have not been fully captured-the lack of diversity within the sample is clearly a potential limitation within our study so we are unable to claim that our results are generalisable to the UK population of people with experience of rare conditions or that we have captured the whole breadth of experiences. Future studies should aim to target underserved communities and collect data in a variety of ways utilising alternative recruitment strategies in order to capture a broader set of experiences of living with a rare condition. However, this exploratory study has produced a robust model which differentiates between variables to best explain overall care experience for the study sample. The variables included in the best fit model correspond well with findings (both quantitative and qualitative) from other rare condition studies [16-21] as well as studies of cancer patients [25] and those in primary care settings [63].

## Conclusions

Our findings indicate the challenges that play the largest part in explaining the varied experiences with rare disease healthcare in the UK for our study respondents. The preponderance of factors relating to care coordination underlines its importance, and related research and service development (for example provision of care coordinators for every affected person) should be supported. The diagnostic odyssey has a significant impact on overall care experience and the needs of those who are likely to never receive a diagnosis should be considered. The other factors that drive overall care experience include healthcare professional awareness, access to a single point of contact, and availability of information and specialist centres.

Significantly, our findings indicate an important potential gap in the Framework: trust and confidence in healthcare professionals. It is possible that if there are improvements across all the priority areas in the Framework then higher levels of trust and confidence in healthcare professionals will follow. However, further research is required to fully understand the foundations of trust and confidence and how this is experienced by all those affected by rare conditions as well as by healthcare professionals. In order to address the bias inherent in many convenience samples, additional effort needs to be focussed on recruiting more diverse samples for research into rare conditions alongside the implementation of a comprehensive national patient registry for rare conditions which would provide valuable data. This could be used to measure the impact of action plans and make policy recommendations which support the broad range of people experiencing rare conditions.

### Supplementary Information

**Additional file 1**. Survey used in the Genetic Alliance 2020 Rare Experience survey.

**Additional file 2**. List of selected variables used in the analysis.

**Additional file 3**. Statistics from selected questions used in the analysis.

## Data Availability

The data that support the findings of this study are available from Genetic Alliance UK, but restrictions apply to the availability of these data, as consent was only obtained for use by the Research Team at Genetic Alliance UK and so are not publicly available. The data in aggregated form are, however, available from the authors upon reasonable request and with the permission of Genetic Alliance UK; please send requests to Amy Hunter amy.hunter@geneticalliance.org.uk.

## Footnotes

1 SWAN UK is a Genetic Alliance UK support group for families with children with ‘Syndromes Without A Name’.

## References

[1] European Commission. EU research on rare diseases 2020. Available from: https://ec.europa.eu/info/research-and-innovation/research-area/health-research-and-innovation/rare-diseases_en.

[2] Nguengang Wakap S, Lambert DM, Olry A, Rodwell C, Gueydan C, Lanneau V (2020). Estimating cumulative point prevalence of rare diseases: analysis of the Orphanet database. Eur J Hum Genet.

[3] Rare Disease UK. What is a rare disease? 2020. Available from: https://www.raredisease.org.uk/what-is-a-rare-disease/.

[4] Dharssi S, Wong-Rieger D, Harold M, Terry S. Review of 11 national policies for rare diseases in the context of key patient needs. Orphanet J Rare Dis. 2017;12. 10.1186/s13023-017-0618-0 PMC5374691 28359278

[5] Forman J, Taruscio D, Llera VA, Barrera LA, Cote TR, Edfjall C (2012). The need for worldwide policy and action plans for rare diseases. Acta Paediatr.

[6] Department of Health and Social Care. The UK Rare Diseases Framework. 2021.

[7] Department of Health. The UK Strategy for Rare Diseases. 2013.

[8] Department of Health and Social Care. The UK Rare Diseases Framework Annex A: results of the national conversation on rare diseases survey 2021. Available from: https://www.gov.uk/government/publications/uk-rare-diseases-framework/the-uk-rare-diseases-framework#:~:text=In%20October%202019%20government%20launched,6%2C293%20responses%20from%20the%20community.

[9] Department of Health and Social Care. England Rare Diseases Action Plan 2022 2022. Available from: https://www.gov.uk/government/publications/england-rare-diseases-action-plan-2022/england-rare-diseases-action-plan-2022#:~:text=UK%20Rare%20Diseases%20Framework,-Development%20of%20the&text=The%204%20priorities%20are%3A,better%20co%2Dordination%20of%20care.

[10] Department of Health and Social Care. England Rare Diseases Action Plan 2023: main report 2023. Available from: https://www.gov.uk/government/publications/england-rare-diseases-action-plan-2023/england-rare-diseases-action-plan-2023-main-report.

[11] NHS Wales Health Collaborative. Wales Rare Diseases Action Plan 2022 - 2026 2022. Available from: https://collaborative.nhs.wales/implementation-groups/rare-diseases/wales-rare-diseases-action-plan-2022-2026/.

[12] Department of Health. Northern Ireland Rare Diseases Action Plan 2022/23 2022. Available from: https://www.health-ni.gov.uk/sites/default/files/publications/health/doh-ni-rare-diseases-action-plan-2223.pdf.

[13] Scottish Government. Rare disease action plan Scotland2022. Available from: https://www.gov.scot/publications/rare-disease-action-plan/.

[14] Genetic Alliance UK. Rare Experience 2020 The lived experiences of people affected by genetic, rare and undiagnosed conditions. Genetic Alliance UK; 2020.

[15] Alexion UK. Reforming Rare Diseases. Alexion UK; 2020.

[16] Courbier S, Berjonneau E. Juggling care and daily life: the balancing act of the rare disease community. Paris, France: EURORDIS; 2017.

[17] Molster C, Urwin D, Di Pietro L, Fookes M, Petrie D, van der Laan S (2016). Survey of healthcare experiences of Australian adults living with rare diseases. Orphanet J Rare Dis.

[18] Kole A, Faurisson F. Rare Diseases Social Epidemiology: Analysis of Inequalities. Rare Diseases Epidemiology. Advances in Experimental Medicine and Biology. 686. Berlin: Springer-Verlag Berlin; 2010. p. 223–50. 10.1007/978-90-481-9485-8_14 20824449

[19] EURORDIS. Improve our experience of health care! Key findings from a survey on patients' and carers' experience of medical care for their rare diseases. EURORDIS - Rare Diseases Europe; 2021.

[20] von der Lippe C, Diesen PS, Feragen KB (2017). Living with a rare disorder: a systematic review of the qualitative literature. Mol Genet Genomic Med.

[21] McMullan J, Crowe AL, Bailie C, Moore K, McMullan LS, Shamandi N (2020). Improvements needed to support people living and working with a rare disease in Northern Ireland: current rare disease support perceived as inadequate. Orphanet J Rare Dis.

[22] McMullan J, Crowe AL, Downes K, McAneney H, McKnight AJ (2022). Carer reported experiences: Supporting someone with a rare disease. Health Soc Care Community.

[23] Kole A, Faurisson F. The voice of 12,000 patients: experiences and expectations of rare disease patients on diagnosis and care in Europe. EURORDIS; 2009.

[24] EURORDIS. INNOVCare Project results demonstrate need for integrated care for rare disease patients EURORDIS; 2018. Available from: https://www.eurordis.org/news/innovcare-project-results-demonstrate-need-integrated-care-rare-disease-patients.

[25] Gomez-Cano M, Lyratzopoulos G, Abel GA (2020). Patient experience drivers of overall satisfaction with care in cancer patients: evidence from responders to the english cancer patient experience survey. J Patient Exp.

[26] Limb L, Nutt S, Sen A (2010). Experience of rare diseases: an insight from patients and families.

[27] Muir E (2016). The Rare Reality—an insight into the patient and family experience of rare disease.

[28] NHS England. Cancer Patient Experience Survey. Available from: https://www.england.nhs.uk/statistics/statistical-work-areas/cancer-patient-experience-survey/.

[29] UCL. CONCORD: CoOrdiNated Care of Rare Diseases 2018. Available from: https://www.ucl.ac.uk/epidemiology-health-care/research/applied-health-research/research/health-care-organisation-and-management-group/concord.

[30] SWAN UK. 2022. Available from: https://www.undiagnosed.org.uk/.

[31] Norman G (2010). Likert scales, levels of measurement and the "laws" of statistics. Adv Health Sci Educ.

[32] Evans WRH (2018). Dare to think rare: diagnostic delay and rare diseases. Br J Gen Pract.

[33] de Vries E, Fransen L, van den Aker M, Meijboom BR (2018). Preventing gatekeeping delays in the diagnosis of rare diseases. Br J Gen Pract.

[34] Yan X, He SJ, Dong D (2020). Determining how far an adult rare disease patient needs to travel for a definitive diagnosis: a cross-sectional examination of the 2018 National Rare Disease Survey in China. Int J Environ Res Public Health.

[35] Huyard C (2009). What, if anything, is specific about having a rare disorder? Patients' judgements on being ill and being rare. Health Expect.

[36] Garrino L, Picco E, Finiguerra I, Rossi D, Simone P, Roccatello D (2015). Living with and treating rare diseases: experiences of patients and professional health care providers. Qual Health Res.

[37] Spencer-Tansley R, Meade N, Ali F, Simpson A, Hunter A (2022). Mental health care for rare disease in the UK - recommendations from a quantitative survey and multi-stakeholder workshop. BMC Health Serv Res.

[38] Frankish N (2022). Good diagnosis improving the experiences of diagnosis for people with rare conditions.

[39] Chao A, Hunter A, Jones J, Ramsey A, Walton H (2021). Barriers and facilitators to diagnosis of rare diseases: a systematic narrative review.

[40] Lewis C, Skirton H, Jones R (2010). Living without a diagnosis: the parental experience. Genet Test Mol Biomark.

[41] Frankish N, Clayton R, Jones J, Simpson A, Hunter A (2023). Delivering a good diagnosis: How to equip healthcare professionals to deliver an improved experience of diagnosis for people living with a rare condition. Orphanet J Rare Dis.

[42] Davlin AS, Clarkin CM, Kalish JM (2018). Beckwith–Wiedemann syndrome: partnership in the diagnostic journey of a rare disorder. Pediatrics.

[43] Simoes E, Sokolov AN, Kronenthaler A, Hiltner H, Schaeffeler N, Rall K (2017). Information ranks highest: Expectations of female adolescents with a rare genital malformation towards health care services. PLoS ONE.

[44] Feragen KB, Rumsey N, Heliovaara A, Boysen BM, Johannessen EC, Havstam C (2017). Scandcleft randomised trials of primary surgery for unilateral cleft lip and Palate: 9. Parental report of social and emotional experiences related to their 5-year-old child's cleft diagnosis. J Plast Surg Hand Surg.

[45] Walton H, Simpson A, Ramsay AIG, Hunter A, Jones J, Ng PL (2022). Development of models of care coordination for rare conditions: a qualitative study. Orphanet J Rare Dis.

[46] Zajac S, Woods A, Tannenbaum S, Salas E, Holladay CL. Overcoming Challenges to Teamwork in Healthcare: A Team Effectiveness Framework and Evidence-Based Guidance. Frontiers in Communication. 2021;6.

[47] Walton H, Simpson A, Ramsay AIG, Hudson E, Hunter A, Jones J (2022). Developing a taxonomy of care coordination for people living with rare conditions: a qualitative study. Orphanet J Rare Dis.

[48] Rare Disease UK (2013). Rare disease care coordination: delivering value, improving services.

[49] Van Groenendael S, Giacovazzi L, Davison F, Holtkemper O, Huang ZX, Wang QY (2015). High quality, patient centred and coordinated care for Alstrom syndrome: a model of care for an ultra-rare disease. Orphanet J Rare Dis.

[50] Goodwin N, Sonola L, Thiel V, Kodner DL (2013). Co-ordinated care for people with complex chronic conditions Key lessons and markers for success.

[51] Simpson A, Bloom L, Fulop NJ, Hudson E, Leeson-Beevers K, Morris S (2021). How are patients with rare diseases and their carers in the UK impacted by the way care is coordinated? An exploratory qualitative interview study. Orphanet J Rare Dis.

[52] Arditi C, Eicher M, Colomer-Lahiguera S, Bienvenu C, Anchisi S, Betticher D, et al. Patients' experiences with cancer care in Switzerland: Results of a multicentre cross-sectional survey. Eur J Cancer Care. 2022:15. 10.1111/ecc.13705 PMC9787424 36130722

[53] Barlow JH, Stapley J, Ellard DR (2007). Living with haemophilia and von Willebrand's: a descriptive qualitative study. Patient Educ Couns.

[54] Grut L, Kvam MH. Facing ignorance: people with rare disorders and their experiences with public health and welfare services. Scandinavian Journal of Disability Research, . 2013;15(1),:pp.20–32.

[55] von der Lippe C, Frich JC, Harris A, Solbraekke KN (2016). Experiences of being heterozygous for Fabry disease: a qualitative study. J Genet Couns.

[56] Whetten K, Leserman J, Whetten R, Ostermann J, Thielman N, Swartz M (2006). Exploring lack of trust in care providers and the government as a barrier to health service use. Am J Public Health.

[57] Schwei RJ, Kadunc K, Nguyen AL, Jacobs EA (2014). Impact of sociodemographic factors and previous interactions with the health care system on institutional trust in three racial/ethnic groups. Patient Educ Couns.

[58] Ferguson E, Dawe-Lane E, Khan Z, Reynolds C, Davison K, Edge D (2022). Trust and distrust: Identifying recruitment targets for ethnic minority blood donors. Transfus Med.

[59] Karnati SA, Wee A, Shirke MM, Harky A (2020). Racial disparities and cardiovascular disease: One size fits all approach?. J Card Surg.

[60] Petersen A (2006). The best experts: the narratives of those who have a genetic condition. Soc Sci Med.

[61] Dures E, Morris M, Gleeson K, Rumsey N (2011). The psychosocial impact of epidermolysis bullosa. Qual Health Res.

[62] Budych K, Helms TM, Schultz C (2012). How do patients with rare diseases experience the medical encounter? Exploring role behavior and its impact on patient-physician interaction. Health Policy.

[63] Paddison CAM, Abel GA, Roland MO, Elliott MN, Lyratzopoulos G, Campbell JL (2015). Drivers of overall satisfaction with primary care: evidence from the English General Practice Patient Survey. Health Expect.

