# Supplementary material for: Factors affecting overall care experience for people living with rare conditions in the UK: exploratory analysis of a quantitative patient experience survey
Source: Orphanet J Rare Dis. 2024 Feb 19;19:77. doi: 10.1186/s13023-024-03081-5 (PMC10877794; doi:10.1186/s13023-024-03081-5)
Supplement: Supplementary file 1 — Additional file 1. Survey used in the Genetic Alliance 2020 Rare Experience survey. [file 13023_2024_3081_MOESM1_ESM.pdf]

GENETIC ALLIANCE UK | RARE DISEASE UK | SWAN UK syndromes without a name

Patient Experience Survey 2020

Welcome

This survey is designed to capture the experience of patients affected by rare and undiagnosed conditions, and their carers. The survey should take approximately 30 minutes to complete.

<u>Who can take part?</u>
You can take part if you are aged 18 or over. You can be:
1. a patient living with a rare or undiagnosed condition;
2. a carer of an adult patient living with a rare or undiagnosed condition, giving the patient's views; or
3. a carer of a patient living with a rare or undiagnosed condition, giving your own views.

Carers can also include parents or relatives. You must live in the UK.

<u>What is the aim of this survey?</u>
Every five years, Genetic Alliance UK collects important information via a patient experience survey. The aim of the survey is to gather and track experiences of large numbers of patients and carers over time. The findings are used to inform our Rare Disease UK campaign. The findings will help us to measure the impact of key policy work (such as the UK Strategy for Rare Diseases), inform the work of the charity and raise awareness of the issues and challenges facing those affected by rare and undiagnosed conditions.

<u>What questions will the survey ask?</u>
The survey will ask for some background information about you/the person you care for. There will then be questions about:
- the search for/finding a diagnosis
- information, awareness and patient voice
- coordination of care
- access to specialist care and treatment
- research
- use of technology
- overall care experience
- impact of Covid-19

At the end of each section, you have the opportunity to provide comments to elaborate on your experience, if you wish.

<u>Who is funding the survey?</u>
This survey is funded by Alexion - a global biopharmaceutical company focused on developing therapies for people living with rare conditions. The survey work (including design, analysis and report writing) will be undertaken by the research team at Genetic Alliance UK.

<u>Who will know I have taken part?</u>
Your responses will be submitted to the research team at Genetic Alliance UK. Genetic Alliance UK will not tell anyone that you have taken part in this survey. Your contact details and responses will be kept securely so that only Genetic Alliance UK team members can access them.

Your personal information will not be passed on to any other third party, although aggregated or summary data will be shared with Alexion. Any personal or identifying information will be removed.

We may use some quotes in future reports, academic publications and/or conferences, but we will not use any names or identifying features in these. However, you should be aware that as with any rare disease research of this nature it is not possible to completely guarantee that you cannot be identified. For that reason, you will be able to decide whether you consent to your quotes being used in this way. Declining will not prevent you from taking part.

All data will be collected and stored in line with the Data Protection Act 2018.

You can read Genetic Alliance UK's privacy policy here.

By taking this survey you consent to the use and storage of your data as described.

* 1. Please state whether you consent to the following: ' I consent to my data being used and stored as described above'

- ◯ Yes
- ◯ No

* 2. Please state whether you consent to the following: ' I consent to my quotes being used in future publications'

- ◯ Yes
- ◯ No

Patient Experience Survey 2020

Section 1: About you/the person you care for

We would like to know a bit more about you/the person you care for.

* 3. Please let us know from whose viewpoint you will be completing this survey. Carers can include patients, relatives and formal carers.

- ◯ I am a patient giving my own views
- ◯ I am a carer supporting an adult patient to share their views
- ◯ I am a carer of a patient and I will be giving my own views

Patient Experience Survey 2020

Section 1: About you

Please tell us about yourself. If you are a patient, or a carer supporting an adult patient to share their views, please provide the patients' details and views only.

4. Where do you (the patient) live?

- ○ East of England
- ○ East Midlands
- ○ London
- ○ North East & Cumbria
- ○ Northern Ireland
- ○ North West of England
- ○ Scotland
- ○ South East of England
- ○ South West of England
- ○ Wales
- ○ West Midlands
- ○ Yorkshire
- ○ Prefer not to say
- ○ Other (please specify)

____

5. What is your sex?

- ○ Male
- ○ Female
- ○ Other
- ○ Prefer not to say

6. How old are you?

- ◯ 18-24
- ◯ 25-34
- ◯ 35-44
- ◯ 45-54
- ◯ 55-64
- ◯ 65-74
- ◯ 75+
- ◯ Prefer not to say

7. What is your ethnic group?

- ◯ White- English/ Welsh/ Scottish/ Northern Irish/ British
- ◯ White- Irish
- ◯ White- Gypsy or Irish Traveller
- ◯ White- Any other White background
- ◯ Mixed/ multiple ethnic groups- White and Black Caribbean
- ◯ Mixed/ multiple ethnic groups- White and Black African
- ◯ Mixed/ multiple ethnic groups- White and Asian
- ◯ Any other mixed/ multiple ethnic background
- ◯ Asian/ Asian British- Indian
- ◯ Asian/ Asian British- Pakistani
- ◯ Asian/ Asian British- Bangladeshi
- ◯ Asian/ Asian British- Any other Asian background
- ◯ Black/ African/ Caribbean/ Black British- African
- ◯ Black/ African/ Caribbean/ Black British- Caribbean
- ◯ Black/ African/ Caribbean/ Black British- Any other Black/ African/ Caribbean background
- ◯ Other ethnic group- Arab
- ◯ Other ethnic group- Any other ethnic group
- ◯ Prefer not to say

8. Do you: (please tick all that apply)

- [ ] Work full time (35 hours per week or more) in paid employment (this may include working from home)
- [ ] Work part time (less than 35 hours per week) in paid employment (this may include working from home)
- [ ] Self employed
- [ ] Do agency/supply work (this may include working from home)
- [ ] Do voluntary work (this may include working from home)
- [ ] Not working or unemployed
- [ ] Recently furloughed as a result of the Covid-19 pandemic
- [ ] Recently made redundant as a result of the Covid-19 pandemic
- [ ] Attend school/college/university (may be currently learning from home)
- [ ] Attend special needs school/college/university (may be currently learning from home)
- [ ] Prefer not to say
- [ ] Other (please specify)

9. Does your rare/undiagnosed condition affect your ability to hold paid employment? (prior to the Covid-19 pandemic)

- ( ) Yes
- ( ) No
- ( ) Don’t know
- ( ) Not applicable

* 10. Does your rare/undiagnosed condition affect your ability to go to school/college/university? (prior to the Covid-19 pandemic)

- ( ) Yes
- ( ) No
- ( ) Don't know
- ( ) Not applicable

Patient Experience Survey 2020

Section 1: About you and the patient you care for

If you are a carer, completing the survey with your own views, please tell us about yourself and the patient you care for.

11. Where do you (the carer) live?

- East of England
- East Midlands
- London
- North East & Cumbria
- Northern Ireland
- North West of England
- Scotland
- South East of England
- South West of England
- Wales
- West Midlands
- Yorkshire
- Prefer not to say
- Other (please specify)

12. What is your (the carer's) sex?

- Male
- Female
- Other
- Prefer not to say

13. How old are you (the carer)?

- 18-24
- 25-34
- 35-44
- 45-54
- 55-64
- 65-74
- 75+
- Prefer not to say

14. What is your (the carer's) ethnic group?

- White- English/ Welsh/ Scottish/ Northern Irish/ British
- White- Irish
- White- Gypsy or Irish Traveller
- White- Any other White background
- Mixed/ multiple ethnic groups- White and Black Caribbean
- Mixed/ multiple ethnic groups- White and Black African
- Mixed/ multiple ethnic groups- White and Asian
- Any other mixed/ multiple ethnic background
- Asian/ Asian British- Indian
- Asian/ Asian British- Pakistani
- Asian/ Asian British- Bangladeshi
- Asian/ Asian British- Any other Asian background
- Black/ African/ Caribbean/ Black British- African
- Black/ African/ Caribbean/ Black British- Caribbean
- Black/ African/ Caribbean/ Black British- Any other Black/ African/ Caribbean background
- Other ethnic group- Arab
- Other ethnic group- Any other ethnic group
- Prefer not to say

15. Do you (the carer): (tick all that apply)

- [ ] Work full time (35 hours or more per week) in paid employment (this may include working from home)
- [ ] Work part time (less than 35 hours per week) in paid employment (this may include working from home)
- [ ] Self employed
- [ ] Do agency/supply work (this may include working from home)
- [ ] Do voluntary work (this may include working from home)
- [ ] Not working or unemployed
- [ ] Recently furloughed as a result of the Covid-19 pandemic
- [ ] Recently made redundant as a result of the Covid-19 pandemic
- [ ] Attend school/college/university (may be currently learning from home)
- [ ] Attend special needs school/college/university (may be currently learning from home)
- [ ] Prefer not to say
- [ ] Other (please specify)

16. Does your caring role affect your ability to hold paid employment? (prior to the Covid-19 pandemic)

- ○ Yes
- ○ No
- ○ Don’t know
- ○ Not applicable

17. Does your caring role affect your ability to go to school/college/university? (prior to the Covid-19 pandemic)

- ○ Yes
- ○ No
- ○ Don't know
- ○ Not applicable

18. Where does the patient live?

- East of England
- East Midlands
- London
- North East & Cumbria
- Northern Ireland
- North West of England
- Scotland
- South East of England
- South West of England
- Wales
- West Midlands
- Yorkshire
- Prefer not to say
- Other (please specify)

19. What is the patient's sex?

- Male
- Female
- Other
- Prefer not to say

20. How old is the patient?

- Under 18
- 18-24
- 25-34
- 35-44
- 45-54
- 55-64
- 65-74
- 75+
- Prefer not to say

21. What is the patient's ethnic group?

- ◯ White- English/ Welsh/ Scottish/ Northern Irish/ British
- ◯ White- Irish
- ◯ White- Gypsy or Irish Traveller
- ◯ White- Any other White background
- ◯ Mixed/ multiple ethnic groups- White and Black Caribbean
- ◯ Mixed/ multiple ethnic groups- White and Black African
- ◯ Mixed/ multiple ethnic groups- White and Asian
- ◯ Any other mixed/ multiple ethnic background
- ◯ Asian/ Asian British- Indian
- ◯ Asian/ Asian British- Pakistani
- ◯ Asian/ Asian British- Bangladeshi
- ◯ Asian/ Asian British- Any other Asian background
- ◯ Black/ African/ Caribbean/ Black British- African
- ◯ Black/ African/ Caribbean/ Black British- Caribbean
- ◯ Black/ African/ Caribbean/ Black British- Any other Black/ African/ Caribbean background
- ◯ Other ethnic group- Arab
- ◯ Other ethnic group- Any other ethnic group
- ◯ Prefer not to say

22. Does the patient: (please tick all that apply)

- [ ] Work full time (35 hours or more) in paid employment (this may include working from home)
- [ ] Work part time (less than 35 hours) in paid employment (this may include working from home)
- [ ] Self employed
- [ ] Do agency/supply work (this may include working from home)
- [ ] Do voluntary work (this may include working from home)
- [ ] Not working or unemployed
- [ ] Recently furloughed as a result of the Covid-19 pandemic
- [ ] Recently made redundant as a result of the Covid-19 pandemic
- [ ] Attend school/college/university (may be currently learning from home)
- [ ] Attend special needs school/college/university (may be currently learning from home)
- [ ] Prefer not to say
- [ ] Other (please specify)

23. Does the rare/undiagnosed condition affect the patient's ability to hold paid employment? (prior to the Covid-19 pandemic)

- ○ Yes
- ○ No
- ○ Don't know
- ○ Not applicable

24. Does the rare/undiagnosed condition affect the patient's ability to go to school/college/university? (prior to the Covid-19 pandemic)

- ○ Yes
- ○ No
- ○ Don't know
- ○ Not applicable

Section 2: About the rare/undiagnosed condition

Please tell us about the condition that you/the person you care for is affected by.

25. Which aspect(s) of health are affected by the rare/undiagnosed condition? (please tick all that apply)

- [ ] Heart, circulatory (Cardiology)
- [ ] Breathing, lungs (Respiratory Medicine)
- [ ] Diabetes, hormones (Endocrinology)
- [ ] Brain, nerves, spinal cord (Neurology)
- [ ] Muscle, ligaments, joints (Rheumatology)
- [ ] Bones, joints (Orthopaedics)
- [ ] Skin (Dermatology)
- [ ] Digestion (Gastroenterology)
- [ ] Kidneys (Nephrology)
- [ ] Mental Health (Psychiatry)
- [ ] Blood (Haematology)
- [ ] Other (please specify)
- [ ] Reproductive
- [ ] Metabolic
- [ ] Chronic pain
- [ ] Vision
- [ ] Hearing
- [ ] Behavioural difficulties
- [ ] Learning difficulties/disabilities
- [ ] Cognitive difficulties
- [ ] Cancer (Oncology)
- [ ] None of the above

* 26. Have you/they been given a definitive diagnosis of a rare condition by a health professional?

(throughout the survey we use the term 'they' to refer to the patient)

- ( ) Yes
- ( ) No
- ( ) Unsure

Patient Experience Survey 2020

Section 2: About the rare condition

27. What is the diagnosed rare condition?

28. What year were you/they diagnosed? Please provide an estimate if you cannot remember the exact year.

* 29. Does the condition have a genetic cause?

- Yes
- No
- Unsure

Section 2: About the rare/undiagnosed condition

30. Do you/they have a condition which is undiagnosed?

- ○ Yes
- ○ No
- ○ Unsure

Patient Experience Survey 2020

Section 3: Diagnosis

Please tell us about your/their experience of searching for a diagnosis.

31. Please state how long it has been since you/they first consulted a doctor about the onset of symptoms.

- ○ Less than 3 months
- ○ 3-6 months
- ○ 6-9 months
- ○ 9 months – 1 year
- ○ 1-2 years
- ○ 2-5 years
- ○ 5-10 years
- ○ 10-20 years
- ○ Over 20 years
- ○ Unsure

32. Have you/they been misdiagnosed in the past?

- ○ Yes
- ○ No
- ○ Unsure

33. If yes, how many times have you/they been misdiagnosed?

- 1
- 2
- 3
- 4
- 5 or more
- Unsure
- I/they have not been misdiagnosed in the past

34. Have you/they been offered genetic or genomic testing?

- Yes
- No
- No, but have sought testing privately
- Unsure

* 35. If yes, have you/they been offered whole genome or exome sequencing? (e.g via NHS, 100,000 Genomes Project or Deciphering Developmental Disorders (DDD) Study)

- Yes
- No
- Unsure
- I/they have not been offered genetic or genomic testing

Section 3: Diagnosis

Please tell us about your/their experience of diagnosis.

36. How long did you/they have to wait for a definitive diagnosis after first consulting a doctor about the onset of symptoms?

- Less than 3 months
- 3-6 months
- 6-9 months
- 9 months – 1 year
- 1-2 years
- 2-5 years
- 5-10 years
- 10-20 years
- Over 20 years
- Unsure
- Diagnosis was made before the onset of condition symptoms e.g. via a genetic test

37. Which healthcare professional made the diagnosis?

- GP
- Doctor who specialises in the rare condition
- Other doctor
- Genetics team
- Other (please specify)

38. Have you/they been misdiagnosed in the past?

- ◯ Yes
- ◯ No
- ◯ Unsure

39. If yes, how many times have you/they been misdiagnosed?

- ◯ 1
- ◯ 2
- ◯ 3
- ◯ 4
- ◯ 5 or more
- ◯ Unsure
- ◯ I/they have not been misdiagnosed in the past

40. Has your/their diagnosis been confirmed by a genetic or genomic test?

- ◯ Yes
- ◯ No
- ◯ Unsure

41. If yes, did you/they receive your/their diagnosis from whole genome or exome sequencing? (e.g. via NHS, the 100,000 Genomes Project or Deciphering Developmental Disorders (DDD) Study)

- ◯ Yes
- ◯ No
- ◯ Unsure
- ◯ I/they have not had their diagnosis confirmed by a genetic or genomic test

Section 3: Diagnosis

42. In your/their search for a diagnosis, how satisfied have you been with the speed at which you were offered genetic or genomic testing?

- ◯ Very satisfied
- ◯ Satisfied
- ◯ Neither satisfied nor unsatisfied
- ◯ Unsatisfied
- ◯ Very unsatisfied
- ◯ Not applicable

43. How would you describe your/their experience of searching for a diagnosis? Please rate on a scale of 1-5.

- ◯ 1 - The NHS never gave up/The NHS has not given up
- ◯ 2
- ◯ 3
- ◯ 4
- ◯ 5 - I feel/felt abandoned by the NHS

44. Is there anything else you would like to say in relation to your/their search for a diagnosis?

You may want to tell us about how the experience of searching for a diagnosis could have been improved, what worked well and/or what didn't work well.

Section 4: Information, awareness and the patient voice

Please tell us about your/their experiences in relation to information, professionals' awareness of the rare/undiagnosed condition and patient involvement.

45. Please indicate how satisfied you were with the information provided by your healthcare professional during the diagnosis period:

If you/they are undiagnosed, please select 'not applicable' at the point of diagnosis and following diagnosis.

	Very satisfied	Satisfied	Neither satisfied nor unsatisfied	Unsatisfied	Very unsatisfied	Not applicable
Before diagnosis	○	○	○	○	○	○
At point of diagnosis	○	○	○	○	○	○
Following diagnosis	○	○	○	○	○	○

46. To what extent do you agree with the following statement: ‘I have sufficient knowledge of my/their rare/undiagnosed condition’

- ○ Strongly agree
- ○ Agree
- ○ Neither agree nor disagree
- ○ Disagree
- ○ Strongly disagree

47. Is there a specific healthcare professional, who you/they can go to with questions about your/their rare/undiagnosed condition?

- ○ Yes
- ○ No
- ○ Unsure

If yes, please specify what type of professional, e.g. GP, nurse

48. If yes, how easy or difficult is it to contact this person?

- ◯ Very easy
- ◯ Quite easy
- ◯ Neither easy nor difficult
- ◯ Quite difficult
- ◯ Very difficult
- ◯ I/they have not tried to contact them yet
- ◯ There is no specific healthcare professional who I/they can go to with questions about the condition

49. Who have been your/their main sources of information and support on the rare/undiagnosed condition? (please tick all that apply)

- ☐ GP
- ☐ Patient organisation
- ☐ A member of the genetics team
- ☐ Other specialist
- ☐ Other families with the condition
- ☐ Online forums which are not related to an organisation (e.g. Facebook groups)
- ☐ Pharmaceutical companies
- ☐ Other (please specify)

50. Is there anything else you would like to say about the information and support you/they have received?

You may want to tell us about how it could be improved, what works well and/or what doesn't work well.

51. To help with our patient group support please tell us whether you understand each of the following terms on a scale from 1 - 5.

	1 - I am confident I understand	2	3	4	5 - I am not at all confident I understand
Genome	○	○	○	○	○
Genome sequencing	○	○	○	○	○
Genome editing	○	○	○	○	○
Gene therapy	○	○	○	○	○
Cell therapy	○	○	○	○	○

Section 4: Information, awareness and the patient voice

52. Are you involved in decisions about your/their treatment?

- Yes, closely involved
- No, not involved
- Not sure/can't remember

53. Are you involved as much as you would like to be in decisions about your/their treatment?

- Yes
- No
- Unsure

54. Is there anything else you would like to say about how you are involved in decision making?

You may want to tell us about how your/their experience could be improved, what works well and/or what doesn't work well.

55. Do you/they have an 'alert card'?

An alert card includes information about the patient's rare disease, any particular aspects of the treatment of their rare disease that needs to be taken into account in providing care to that patient, and details of how to contact an individual expert in that patient's care. It can be used in emergency situations.

- Yes
- No
- Unsure

56. To what extent do you agree with the following statement for each of the scenarios below:
'I have confidence and trust in the professionals treating me/the person I care for'

	Strongly agree	Agree	Neither agree nor disagree	Disagree	Strongly disagree	Not applicable
Hospital staff involved in ongoing care	○	○	○	○	○	○
Staff at the local general practice	○	○	○	○	○	○
Paramedics and staff in Emergency Departments	○	○	○	○	○	○
Professionals working in social care	○	○	○	○	○	○

57. To what extent do you agree with the following statement for each of the scenarios below:
'Professionals have sufficient information about the condition'

(if you/they do not have a diagnosed condition, please state the extent to which you agree that professionals are sufficiently informed about your/their specific symptoms).

	Strongly agree	Agree	Neither agree nor disagree	Disagree	Strongly disagree	Not applicable
Hospital staff involved in ongoing care	○	○	○	○	○	○
Staff at the local general practice	○	○	○	○	○	○
Paramedics and staff in Emergency Departments	○	○	○	○	○	○
Professionals working in social care	○	○	○	○	○	○

58. Is there anything else you would like to say about the awareness of rare/undiagnosed conditions amongst professionals?

You may want to tell us about how your/their experience could be improved, what works well and/or what doesn't work well.

Section 5: Coordination of Care

Please tell us about your/their experiences of care coordination.

59. How frequently do you/they use health services in relation to your/their rare/undiagnosed condition? Please estimate the number of visits you/they have had with a healthcare professional in the 12 months prior to March 2020 (i.e. pre Covid-19). Visits can include clinics at hospitals, appointments with your GP practice, home care visits, visits to emergency departments and/or admissions to hospital.

60. How many different types of clinics do you/they currently attend for your/their rare/undiagnosed condition?

- None
- 1-2
- 3-4
- 5-6
- More than 6
- Unsure

61. Approximately, how long does it take you/them to travel to the clinic which is furthest from your/their home?

- Up to 1 hour
- 1-2 hours
- 2-3 hours
- 3-4 hours
- 4-5 hours
- Over 5 hours
- Unsure
- I/they do not attend any clinics for my/their rare/undiagnosed condition

62. Do you/they receive some or all of your care in a different nation to the one that you/they live in? (e.g. you live in Wales, but you travel to receive care in England)

- ◯ Yes
- ◯ No
- ◯ Unsure

* 63. Who coordinates (or organises) the majority of your/their care?

- ◯ We do (the patient and/or carer)
- ◯ A dedicated care coordinator *(a professional with a recognised role in helping patients and carers manage a range of needs between different professionals or across care settings. They may be a full-time coordinator or may coordinate care as part of their main role, such as a GP).*
- ◯ Shared responsibility across professionals with no single coordinator
- ◯ Unsure
- ◯ Other (please specify)

Section 5: Coordination of care

64. When were you/they first given the support of a dedicated care coordinator?

- Before diagnosis
- At the time of diagnosis
- After diagnosis
- Unsure
- I/they have not been diagnosed

65. Who is your/their dedicated care coordinator?

- Someone who is employed specifically as a care coordinator
- GP
- Hospital doctor
- Community paediatrician
- Representative of patient group or charity
- Specialist nurse
- Administrator
- Other (please specify)

Section 5: Coordination of care

66. Ideally, who would you like to coordinate your/their care?

- ◯ Patient and/or carer
- ◯ Someone who is employed specifically as a care coordinator
- ◯ Shared responsibility across professionals with no single coordinator
- ◯ GP
- ◯ Hospital doctor
- ◯ Community paediatrician
- ◯ Representative of patient group or charity
- ◯ Specialist nurse
- ◯ Administrator
- ◯ Other (please specify)

Section 5: Coordination of Care

67. Do you/they have a care plan?

A care plan is a paper or electronic document which describes the health services and support that are needed and should be agreed between patients, carers and professionals. The care plan may be a single document or it may be part of another record which includes non-health services such as an Educational Care and Health Plan (ECHP).

- ◯ Yes
- ◯ No
- ◯ Unsure

68. Do you feel that your/their care is coordinated effectively?

- ◯ Yes
- ◯ No
- ◯ Unsure

69. To what extent do you agree with the following statements:

	Strongly agree	Agree	Neither agree nor disagree	Disagree	Strongly disagree	Not applicable
The professionals providing my/their care work as a team	◯	◯	◯	◯	◯	◯
The timing and frequency of my/their appointments are convenient for the patient/carer/family	◯	◯	◯	◯	◯	◯
I/they would prefer more appointments to be provided locally	◯	◯	◯	◯	◯	◯

70. If you/they have experience of transitioning from paediatric to adult services, how satisfied were you with the transition process?

- Very satisfied
- Satisfied
- Neither satisfied nor unsatisfied
- Unsatisfied
- Very Unsatisfied
- No experience of transitioning from paediatric to adult services

71. Is there anything else you would like to say about how your/their care is coordinated or organised?

You may want to tell us about how your/their experience could be improved, what works well and/or what doesn't work well.

Section 6: Access to specialist care and treatments

Please tell us about your/their experience in relation to specialist care and access to treatments.

72. Do you/they have a doctor who is an expert in your/their rare condition?

- ◯ Yes
- ◯ No
- ◯ Unsure

* 73. Do you know if there is a specialist centre for your/their condition?

A 'specialist centre' is a centre that is able to provide expert advice on diagnosis, assessment and treatment of a particular condition. The centre will be made up of a team of different specialists, sometimes also including scientists and researchers. Specialist centres support patients across the UK, not just in their local area.

- ◯ Yes there is
- ◯ No there is not
- ◯ Unsure

Section 6: Access to specialist care and treatments

74. Do you/they access the specialist centre for the condition?

- ○ Yes
- ○ No
- ○ Don’t know

75. If you answered yes, what does the specialist centre do well and is there anything you think the specialist centre should do, which it does not do at the moment?

76. If you answered no, please can you explain why you/they do not access the specialist centre for the condition.

Section 6: Access to specialist care and treatments

* 77. Are you aware of one or more medication(s) for your/their condition? *(We are interested in medicines that are specific to one or a small number of rare diseases)*

- ○ Yes I am aware of one
- ○ Yes I am aware of more than one
- ○ No
- ○ Unsure

Section 6: Access to specialist care and treatments

78. Please tell us the names of up to 3 medications for your/their rare condition

Name of medication 1

Name of medication 2

Name of medication 3

79. Please tell us more about medication 1.

	Yes	No	Unsure	Not applicable
Do you/they receive the medication?	○	○	○	○
If you/they do not receive the medication, has your/their doctor tried to access it for you/them?	○	○	○	○
If you/they do not receive the medication, are you aware of other patients in the UK, with the same rare condition, who do receive the medication?	○	○	○	○

80. If applicable, please tell us more about medication 2.

	Yes	No	Unsure	Not applicable
Do you/they receive the medication?	○	○	○	○
If you/they do not receive the medication, has your/their doctor tried to access it for you/them?	○	○	○	○
If you/they do not receive the medication, are you aware of other patients in the UK, with the same rare condition, who do receive the medication?	○	○	○	○

81. If applicable, please tell us more about medication 3.

	Yes	No	Unsure	Not applicable
Do you/they receive the medication?	○	○	○	○
If you/they do not receive the medication, has your/their doctor tried to access it for you/them?	○	○	○	○
If you/they do not receive the medication, are you aware of other patients in the UK, with the same rare condition, who do receive the medication?	○	○	○	○

Section 6: Access to specialist care and treatments

82. Are you aware of a medicine in development for your/their condition?

- ◯ Yes
- ◯ No
- ◯ Unsure

If yes, how many?

[ ]

83. To what extent do you agree with the following statements about decisions on medicines for rare conditions for your country?

	Strongly agree	Agree	Neither agree nor disagree	Disagree	Strongly disagree
I am satisfied with the process used to decide on funding rare condition medications in the NHS	◯	◯	◯	◯	◯
Enough money is allocated to rare disease medicines	◯	◯	◯	◯	◯
Decisions on funding and pricing of medicines are transparent	◯	◯	◯	◯	◯
The system is unfair on people living with rare conditions	◯	◯	◯	◯	◯
The system is too slow to make decisions	◯	◯	◯	◯	◯

84. Is there anything else you would like to say about accessing specialist care, medicines or other treatments (e.g. surgery) for your/their rare/undiagnosed condition?

You may want to tell us about how your/their experience could be improved, what works well and/or what doesn't work well.

Patient Experience Survey 2020

Section 7: Research

Please tell us about your/their experience in relation to research.

85. Have you/they participated in any of the following in the last 5 years related to your/their condition? (please tick all that apply):

- ☐ Sharing information with/joining a condition-specific patient registry
- ☐ Sharing information with/joining the National Congenital Anomaly and Rare Disease Registration Service (NCARDRS)
- ☐ Identifying research priorities and/or designing research studies
- ☐ Taken part in a clinical trial(s) (e.g. for drugs or other treatments)
- ☐ As a patient representative (e.g. on the ethics board for a trial or a research project steering/advisory group)
- ☐ Biobanking (e.g. providing tissue/DNA/blood samples for research)
- ☐ Genomic research (e.g. 100,000 Genomes Project, gene editing trials)
- ☐ Recruiting patients into trials
- ☐ Qualitative/patient experience research (e.g. surveys, interviews)
- ☐ I/they have not participated in research
- ☐ Other (please specify)

86. To what extent do you agree with the following statement? 'I would like more opportunities for me/them to take part in research in the future'

- ○ Strongly agree
- ○ Agree
- ○ Neither agree nor disagree
- ○ Disagree
- ○ Strongly disagree

87. Is there anything else you want to say in relation to research?

You may want to tell us about how your/their experience in relation to research could be improved, what works well and/or what doesn't work well.

Patient Experience Survey 2020

Section 8: Use of technology

Please tell us about your/their experience of using technology to improve the care and management of the rare/undiagnosed condition.

88. Have you/they ever been offered or used any of the following, and if so, how useful have you found them?

	1 - Not useful at all	2	3	4	5 - Very useful	Not used/offered
Consultations/appointments with professionals via video conferencing	○	○	○	○	○	○
Consultations/appointments with professionals via phone	○	○	○	○	○	○
Website (used by patient or carer) to store and share medical information	○	○	○	○	○	○
Smartphone applications designed to help the patient or carer manage the rare condition	○	○	○	○	○	○
Online messaging or discussion boards with professionals	○	○	○	○	○	○

89. Are there any other ways that you/they have used technology, which have improved your/their care experience? (e.g. use of wearables) If so, please provide details.

90. Has the use of technology had a negative impact on your/their care experience? If so, please provide details.

Section 9: Overall care

In this section, please tell us about your overall care experience. The questions ask you to reflect on general experiences. Please think about your overall experience in the past five years.

91. Overall, how would you rate the care you/they receive for their rare/undiagnosed condition? Please rate on a scale of 1-5.

- ◯ 1 - very poor
- ◯ 2
- ◯ 3
- ◯ 4
- ◯ 5 - very good

92. Have you noticed any changes to your/their experience of care in the past five years?

- ◯ Yes, the quality of care has improved
- ◯ Yes, the quality of care has got worse
- ◯ There has been no change
- ◯ Not applicable

93. Please explain your answer.

94. Are you aware of the UK Rare Disease Strategy?

- ◯ Yes
- ◯ No
- ◯ Unsure

Section 10: Impact of Covid-19 on your experiences

Please think about your experience more recently...

95. To what extent has the Covid-19 pandemic changed your/their experience of care with a rare/undiagnosed condition? Please rate on a scale of 1 - 5.

- 1- It has not changed at all
- 2
- 3
- 4
- 5 - It has changed alot

96. Please explain your answer.

97. Since the beginning of the Covid-19 pandemic, have you/they experienced an interruption in the care you/they normally receive for the rare/undiagnosed condition?

- Yes, care has been interrupted to a large extent
- Yes, care has been interrupted to a small extent
- No, care has not changed at all
- Unsure

98. Have you/they chosen to miss appointments because of concerns about going to the hospital?

- ◯ Yes
- ◯ No
- ◯ Unsure

99. Since the beginning of the Covid-19 pandemic, have any of your/their usual appointments been provided virtually?

- ◯ Yes
- ◯ No
- ◯ Unsure/not applicable

100. If yes, how satisfied have you been with accessing care in this way?

- ◯ Very satisfied
- ◯ Satisfied
- ◯ Neither satisfied nor unsatisfied
- ◯ Unsatisfied
- ◯ Very unsatisfied
- ◯ Not applicable/ I/they have not had any appointments virtually

101. How satisfied have you been with the information and support provided to them/you during the Covid-19 pandemic?

	Very satisfied	Satisfied	Neither satisfied nor unsatisfied	Unsatisfied	Very unsatisfied	Not applicable
Government	◯	◯	◯	◯	◯	◯
Charities/support groups	◯	◯	◯	◯	◯	◯
Healthcare professionals involved in your/their care	◯	◯	◯	◯	◯	◯

102. Is there anything else you would like to say about your/their experience during the Covid-19 pandemic?

Final Questions

* 103. Would you be happy for the Rare Disease UK team at Genetic Alliance UK to contact you in the future with information on any of the following:

	Yes	No
future workshops, associated with this survey	◯	◯
sharing your story for our influencing / policy work	◯	◯
getting involved with other research projects we are involved with	◯	◯
fundraising opportunities to support our work	◯	◯
opportunities to share your experience with the media	◯	◯

* 104. Would you be happy to be added to our Rare Disease UK mailing list to stay informed about our work?

◯ Yes

◯ No

105. If you selected ‘yes’ for any of the above, please provide your email address:

Patient Experience Survey 2020

Thank you

The survey is now complete. Thank you for taking the time to share your views. If you have any questions about this survey please contact amy.hunter@geneticalliance.org.uk
