# Supplementary material for: Factors affecting overall care experience for people living with rare conditions in the UK: exploratory analysis of a quantitative patient experience survey
Source: Orphanet J Rare Dis. 2024 Feb 19;19:77. doi: 10.1186/s13023-024-03081-5 (PMC10877794; doi:10.1186/s13023-024-03081-5)
Supplement: Supplementary file 2 — Additional file 2. List of selected variables used in the analysis. [file 13023_2024_3081_MOESM2_ESM.pdf]

Additional File 2: List of selected variables, question wording and variable type

Variable by survey section	Question wording	Variable type – test applied
**Section 1: Demographics**		
Age of patient	6. How old are you? /<br>20. How old is the patient?	Ordinal data - Kendall's Tau-b
Child/Adult indicator	Variable created from Q6 and Q20<br>Child is under 18, Adult is 18 and over	Nominal data (2 categories – child / adult) - Kendall's Tau-b
Sex of patient	5. What is your sex? /<br>19. What is the patient's sex?	Nominal data (2 categories – female / male) - Kendall's Tau-b
Respondent's location (UK Nation - excluding NI as numbers are too small)	Variable created from Q4<br>4. Where do you (the patient) live?	Nominal variable - Chi-squared
Patient's ability to work affected	9. Does your rare/undiagnosed condition affect your ability to hold paid employment? (prior to the Covid-19 pandemic) /<br>23. Does the rare/undiagnosed condition affect the patient's ability to hold paid employment? (prior to the Covid-19 pandemic)	Nominal data (2 categories – yes / no) - Kendall's Tau-b
Patient's ability to study affected	10. Does your rare/undiagnosed condition affect your ability to go to school/college/university? (prior to the Covid-19 pandemic)<br>24. Does the rare/undiagnosed condition affect the patient's ability to go to school/college/university? (prior to the Covid-19 pandemic)	Nominal data (2 categories – yes / no) - Kendall's Tau-b
**Section 2: About the rare / undiagnosed condition**		
Count of number of aspects of health mentioned	25. Which aspect(s) of health are affected by the rare/undiagnosed condition?<br>Variable created from count of aspects of health affected mentioned in Q25	Discrete variable - Kruskal-Wallis
Condition Complexity	Grouped variable created from count of number of aspects of health affected (one or less, 2-3, 4-5, 6-7, 8+)	Ordinal data - Kendall's Tau-b
Whether or not diagnosed	26. Have you/they been given a definitive diagnosis of a rare condition by a health professional? /<br>30. Do you/they have a condition which is undiagnosed?	Nominal data (2 categories – diagnosed / undiagnosed) - Kendall's Tau-b
Number of years since diagnosis	28. What year were you/they diagnosed?<br>A variable of grouped number of years was created (less than 1 year, 1-2 years, 3-5 years, 6-10 years, 11-20 years, more than 20 years)	Ordinal data - Kendall's Tau-b

Variable by survey section	Question wording	Variable type – test applied
**Section 3: Diagnosis**		
Whether have been misdiagnosed	32. Have you/they been misdiagnosed in the past? *(those without a diagnosis)*<br>38. Have you/they been misdiagnosed in the past? *(those with a diagnosis)*	Nominal data (2 categories – yes / no) - Kendall's Tau-b
Number of times misdiagnosed	33. If yes, how many times have you/they been misdiagnosed? *(asked of those without a diagnosis)*<br>39. If yes, how many times have you/they been misdiagnosed? *(asked of those with a diagnosis)*	Ordinal data - Kendall's Tau-b
Length of wait for definitive diagnosis	36. How long did you/they have to wait for a definitive diagnosis after first consulting a doctor about the onset of symptoms? (categories less than 1 year combined together)	Ordinal data - Kendall's Tau-b
Which healthcare professional made the diagnosis	37. Which healthcare professional made the diagnosis?	Nominal variable - Chi-squared
Experience of searching for a diagnosis	43. How would you describe your/their experience of searching for a diagnosis? Please rate on a scale of 1-5	Ordinal data - Kendall's Tau-b
**Section 4: Information, awareness and the patient voice**		
Satisfaction with information provided – before diagnosis	45. Please indicate how satisfied you were with the information provided by your healthcare professional during the diagnosis period: Before diagnosis	Ordinal data - Kendall's Tau-b
Satisfaction with information provided – at diagnosis	45. Please indicate how satisfied you were with the information provided by your healthcare professional during the diagnosis period: At point of diagnosis	Ordinal data - Kendall's Tau-b
Satisfaction with information provided – following diagnosis	45. Please indicate how satisfied you were with the information provided by your healthcare professional during the diagnosis period: Following diagnosis	Ordinal data - Kendall's Tau-b
Agreement with statement ' I have sufficient information about condition'	46. To what extent do you agree with the following statement: 'I have sufficient knowledge of my/their rare/undiagnosed condition'	Ordinal data - Kendall's Tau-b
Is there a specific heath care professional to contact about the condition?	47. Is there a specific healthcare professional, who you/they can go to with questions about your/their rare/undiagnosed condition?	Nominal data (2 categories – yes / no) - Kendall's Tau-b
If there is a specific healthcare professional to contact, how easy is it to contact them	48. If yes, how easy or difficult is it to contact this person?	Ordinal data - Kendall's Tau-b

Variable by survey section	Question wording	Variable type – test applied
Involved in decision about treatment?	52. Are you involved in decisions about your/their treatment?	Nominal data (2 categories yes / no) - Kendall's Tau-b
Whether or not have an alert card	55. Do you/they have an 'alert card'?	Nominal data (2 categories yes / no) - Kendall's Tau-b
Trust and confidence in Hospital staff	56. To what extent do you agree with the following statement for each of the scenarios below: 'I have confidence and trust in the professionals treating me/the person I care for': Hospital staff involved in ongoing care	Ordinal data - Kendall's Tau-b
Trust and confidence in GP staff	56. To what extent do you agree with the following statement for each of the scenarios below: 'I have confidence and trust in the professionals treating me/the person I care for': Staff at the local general practice	Ordinal data - Kendall's Tau-b
Trust and confidence in ED staff and Paramedics	56. To what extent do you agree with the following statement for each of the scenarios below: 'I have confidence and trust in the professionals treating me/the person I care for': Paramedics and staff in Emergency Departments	Ordinal data - Kendall's Tau-b
Trust and confidence in Social care staff	56. To what extent do you agree with the following statement for each of the scenarios below: 'I have confidence and trust in the professionals treating me/the person I care for': Professionals working in social care	Ordinal data - Kendall's Tau-b
Hospital staff are sufficiently informed about condition	57. To what extent do you agree with the following statement for each of the scenarios below: 'Professionals have sufficient information about the condition': Hospital staff involved in ongoing care	Ordinal data - Kendall's Tau-b
GP staff are sufficiently informed about condition	57. To what extent do you agree with the following statement for each of the scenarios below: 'Professionals have sufficient information about the condition': Staff at the local general practice	Ordinal data - Kendall's Tau-b
Paramedics are sufficiently informed about condition	57. To what extent do you agree with the following statement for each of the scenarios below: 'Professionals have sufficient information about the condition': Paramedics and staff in Emergency Departments	Ordinal data - Kendall's Tau-b
Social care staff are sufficiently informed about condition	57. To what extent do you agree with the following statement for each of the scenarios below: 'Professionals have sufficient information about the condition': Professionals working in social care	Ordinal data - Kendall's Tau-b

Variable by survey section	Question wording	Variable type – test applied
**Section 5: Coordination of care**		
Number of times had contact with Health Service (Grouped)	59. How frequently do you/they use health services in relation to your/their rare/undiagnosed condition? Variable with grouped categories created (0, 1-2, 3-5, 6-10, 11-20, 21 or more)	Ordinal data - Kendall's Tau-b
Number of different clinics attend	60. How many different types of clinics do you/they currently attend for your/their rare/undiagnosed condition? [categories 5-6 and more than 6 were combined]	Ordinal data - Kendall's Tau-b
Time to travel to furthest clinic	61. Approximately, how long does it take you/them to travel to the clinic which is furthest from your/their home?	Ordinal data - Kendall's Tau-b
Who coordinates care?	63. Who coordinates (or organises) the majority of your/their care?	Nominal variable - Chi-squared
Whether have a care plan	67. Do you/they have a care plan?	Nominal data (2 categories – yes / no) - Kendall's Tau-b
Feel that care is coordinated effectively	68. Do you feel that your/their care is coordinated effectively?	Nominal data (2 categories – yes / no) - Kendall's Tau-b
Agreement with: The professionals providing care work as a team	69. To what extent do you agree with the following statements:<br>The professionals providing my/their care work as a team	Ordinal data - Kendall's Tau-b
Agreement with: The timing and frequency of appointments are convenient	69. To what extent do you agree with the following statements:<br>The timing and frequency of my/their appointments are convenient for the patient/carer/family	Ordinal data - Kendall's Tau-b
Agreement with: Would prefer more appointments to be provided locally	69. To what extent do you agree with the following statements:<br>I/they would prefer more appointments to be provided locally	Ordinal data - Kendall's Tau-b
**Section 6: Access to specialist care and treatments**		
Do they have a doctor who is an expert?	72. Do you/they have a doctor who is an expert in your/their rare condition?	Nominal data (2 categories – yes / no) - Kendall's Tau-b
Whether there is a specialist centre	73. Do you know if there is a specialist centre for your/their condition? (Yes, No, Unsure)	Nominal variable - Chi-squared
If so whether they access the specialist centre	74. Do you/they access the specialist centre for the condition?	Nominal data (2 categories – yes / no) - Kendall's Tau-b
**Section 9: Overall care**		
Changes in quality of care in past 5 years	92. Have you noticed any changes to your/their experience of care in the past five years? (Yes, the quality of care has improved / There has been no change / Yes, the quality of care has got worse)	Ordinal data - Kendall's Tau-b

No variables / questions were selected from sections 7, 8 and 10 from the survey
