# Supplementary material for: Factors affecting overall care experience for people living with rare conditions in the UK: exploratory analysis of a quantitative patient experience survey
Source: Orphanet J Rare Dis. 2024 Feb 19;19:77. doi: 10.1186/s13023-024-03081-5 (PMC10877794; doi:10.1186/s13023-024-03081-5)
Supplement: Supplementary file 3 — Additional file 3. Statistics from selected questions used in the analysis. [file 13023_2024_3081_MOESM3_ESM.pdf]

Additional File 3 – Selected descriptive statistics from the Genetic Alliance UK 2020 Rare Experience survey

Q91. Overall, how would you rate the care you/they receive for their rare/undiagnosed condition? Please rate on a scale of 1-5.

	Number of respondents	Valid percent
**1 – very poor**	177	18.5
**2**	176	18.4
**3**	265	27.7
**4**	198	20.7
**5 – very good**	139	14.6
**Missing**	65	

Q43. How would you describe your/their experience of searching for a diagnosis? Please rate on a scale of 1-5.

	Number of respondents	Valid percent
**1 – The NHS never gave up / The NHS has not given up**	236	23.5
**2**	142	14.1
**3**	217	21.6
**4**	138	13.7
**5 – I feel / felt abandoned by the NHS**	272	27.1
**Missing**	15	

45. Please indicate how satisfied you were with the information provided by your healthcare professional during the diagnosis period:

Before diagnosis	Number of respondents	Valid percent
**Very satisfied**	108	11.9
**Satisfied**	174	19.1
**Neither satisfied or unsatisfied**	180	19.8
**Unsatisfied**	181	19.9
**Very unsatisfied**	266	29.3
**Not applicable / missing**	111	

At point of diagnosis	Number of respondents	Valid percent
**Very satisfied**	221	24.4
**Satisfied**	313	34.5
**Neither satisfied or unsatisfied**	145	16.0
**Unsatisfied**	134	14.8
**Very unsatisfied**	93	10.3
**Not applicable / missing**	114	

After diagnosis	Number of respondents	Valid percent
**Very satisfied**	203	22.4
**Satisfied**	243	26.8
**Neither satisfied or unsatisfied**	155	17.1
**Unsatisfied**	155	17.1
**Very unsatisfied**	151	16.6
**Not applicable / missing**	113	

47. Is there a specific healthcare professional, who you/they can go to with questions about your/their rare/undiagnosed condition?

	Number of respondents	Valid percent
**No**	347	36.9
**Yes**	594	63.1
**Unsure / missing**	79	

48. If yes, how easy or difficult is it to contact this person? (Only applicable to those respondents who at Q47 said there was a specific healthcare professional who they could go to with questions)

	Number of respondents	Valid percent
**Very difficult**	37	6.3
**Quite difficult**	108	18.4
**Neither easy nor difficult**	69	11.7
**Quite easy**	223	37.9
**Very easy**	130	22.1
**Not tried to contact**	21	3.6
**Not applicable / missing**	432	

69. To what extent do you agree with the following statements:

The professionals providing my/their care work as a team	Number of respondents	Valid percent
**Strongly disagree**	188	21.5
**Disagree**	200	22.9
**Neither agree nor disagree**	190	21.7
**Agree**	192	21.9
**Strongly agree**	105	12.0
**Not applicable / missing**	145	

69. To what extent do you agree with the following statements:

The timing and frequency of my/their appointments are convenient for the patient/carer/family	Number of respondents	Valid percent
**Strongly disagree**	110	12.2
**Disagree**	179	19.9
**Neither agree nor disagree**	237	26.4
**Agree**	267	29.7
**Strongly agree**	106	11.8
**Not applicable / missing**	121	

60. How many different types of clinics do you/they currently attend for your/their rare/undiagnosed condition?

	Number of respondents	Valid percent
**None**	187	18.4
**1 - 2**	431	42.4
**3 - 4**	245	24.1
**5 - 6**	86	8.5
**More than 6**	55	5.4
**Unsure**	13	1.3
**Missing**	3	

68. Do you feel that your/their care is coordinated effectively?

	Number of respondents	Valid percent
**No**	499	50.2
**Yes**	296	29.7
**Unsure**	200	20.1
**Missing**	25	

73. Do you know if there is a specialist centre for your/their condition?

	Number of respondents	Valid percent
**No there is not**	209	20.5
**Yes there is**	489	47.9
**Unsure**	322	31.6
**Missing**	0	

74. Do you/they access the specialist centre for the condition? (Only asked of those respondents who answered yes at Q73)

	Number of respondents	Valid percent
**No**	208	42.8
**Yes**	253	52.1
**Don't know**	25	5.1
**Not applicable / missing**	534	

56. To what extent do you agree with the following statement for each of the scenarios below: 'I have confidence and trust in the professionals treating me/the person I care for'

Hospital staff involved in ongoing care	Number of respondents	Valid percent
**Strongly disagree**	113	12.1
**Disagree**	128	13.7
**Neither agree nor disagree**	143	15.3
**Agree**	308	32.9
**Strongly agree**	245	26.1
**Not applicable / missing**	83	
